# *Plasmodium malariae* and *P. ovale* genomes provide insights into malaria parasite evolution

**DOI:** 10.1038/nature21038

**Published:** 2017-01-25

**Authors:** Gavin G. Rutledge, Ulrike Böhme, Mandy Sanders, Adam J. Reid, James A. Cotton, Oumou Maiga-Ascofare, Abdoulaye A. Djimdé, Tobias O. Apinjoh, Lucas Amenga-Etego, Magnus Manske, John W. Barnwell, François Renaud, Benjamin Ollomo, Franck Prugnolle, Nicholas M. Anstey, Sarah Auburn, Ric N. Price, James S. McCarthy, Dominic P. Kwiatkowski, Chris I. Newbold, Matthew Berriman, Thomas D. Otto

**Affiliations:** 1 Wellcome Trust Sanger Institute, Hinxton, Cambridge CB10 1SA, UK; 2 Malaria Research and Training Center, University of Science, Techniques, and Technologies of Bamako, Bamako, BP E.2528, Mali; 3 German Center for Infection Research, 20359 Hamburg, Germany; 4 University of Buea, Post Office Box 63, Buea, South West Region, Republic of Cameroon; 5 Navrongo Health Research Centre, Post Office Box 114, Navrongo, Upper East Region, Ghana; 6 Centers for Disease Control and Prevention, Atlanta, Georgia 30333, USA; 7 Laboratoire MIVEGEC (UM1-CNRS-IRD), 34394 Montpellier, France; 8 Centre International de Recherches Médicales de Franceville, BP 709 Franceville, Gabon; 9 Global and Tropical Health Division, Menzies School of Health Research and Charles Darwin University, Darwin, Northern Territory 0810, Australia; 10 Centre for Tropical Medicine and Global Health, Nuffield Department of Clinical Medicine, University of Oxford, Oxford OX3 7LJ, UK; 11 Clinical Tropical Medicine Laboratory, QIMR Berghofer Medical Research Institute, University of Queensland, Brisbane, Queensland 4006, Australia; 12 Wellcome Trust Centre for Human Genetics, University of Oxford, Oxford OX3 7BN, UK; 13 Weatherall Institute of Molecular Medicine, University of Oxford, John Radcliffe Hospital, Oxford OX3 9DS, UK

## Abstract

Elucidation of the evolutionary history and interrelatedness of *Plasmodium* species that infect humans has been hampered by a lack of genetic information for three human-infective species: *P. malariae* and two *P. ovale* species (*P. o. curtisi* and *P. o. wallikeri*)^1^. These species are prevalent across most regions in which malaria is endemic^2^,^3^ and are often undetectable by light microscopy^4^, rendering their study in human populations difficult^5^. The exact evolutionary relationship of these species to the other human-infective species has been contested^6^,^7^. Using a new reference genome for *P. malariae* and a manually curated draft *P. o. curtisi* genome, we are now able to accurately place these species within the *Plasmodium* phylogeny. Sequencing of a *P. malariae* relative that infects chimpanzees reveals similar signatures of selection in the *P. malariae* lineage to another *Plasmodium* lineage shown to be capable of colonization of both human and chimpanzee hosts. Molecular dating suggests that these host adaptations occurred over similar evolutionary timescales. In addition to the core genome that is conserved between species, differences in gene content can be linked to their specific biology. The genome suggests that *P. malariae* expresses a family of heterodimeric proteins on its surface that have structural similarities to a protein crucial for invasion of red blood cells. The data presented here provide insight into the evolution of the *Plasmodium* genus as a whole.

A reference genome of *P. malariae* was produced from clinically isolated parasites and sequenced using long-read sequencing technology (Table 1; Supplementary Information). The assembly surpasses available draft genome data for *P. malariae*^7^, especially in terms of contiguity (63 versus 7,270 scaffolds, N50 = 2.3 Mb versus 6.4 kb) (Supplementary Information; Extended Data Table 1) allowing large-scale structural changes to be accurately determined. Against a background of near-complete collinearity with *P. vivax,* we found a previously undescribed large reciprocal translocation between chromosomes 6 and 10 and a pericentric inversion of chromosome 5 (Extended Data Fig. 1a, b). Additional draft genomes for both species of *P. ovale* were assembled from *P. falciparum* co-infections and the genome of a parasite that we call ‘*P. malariae*-like’ was assembled from a chimpanzee co-infected with *P. reichenowi* (Fig. 1; Table 1; Extended Data Table 2; Supplementary Information).

To investigate host-specific adaptation of parasites to human and chimpanzee hosts, we compared *P. malariae* to *P. malariae-like.* We found lower levels of nucleotide diversity in the human-infective species than in the chimpanzee-infective species (Table 1; Extended Data Fig. 2a). This mirrors the lower levels of nucleotide diversity in the human parasite *P. falciparum* than in its chimpanzee-infective relative *P. reichenowi*^8^. In both cases, the lack of diversity in human-infective species suggests recent population expansions. However, we found that a species that infects New World primates termed *P. brasilianum* was indistinguishable from *P. malariae* (Extended Data Fig. 2b), as previously suggested^9^. Thus host adaptation in the *P. malariae* lineage appears to be less restricted than in *P. falciparum*.

Using additional samples to calculate standard measures of molecular evolution (Methods; Supplementary Information), we identified a subset of genes under selection in both *P. malariae* and *P. malariae-like* and in an earlier study of *P. falciparum* and *P. reichenowi*^10^ (Extended Data Fig. 3a; Extended Data Table 3), showing some conservation of selection pressures in *Plasmodium* lineages and suggesting host-specific adaptation of parasites to human and chimpanzee hosts. There is evidence that five genes are under diversifying selection in both lineages (Extended Data Fig. 3b), including one encoding Merozoite surface protein 1 (*msp1).* Two genes expressed in blood stages are under significant balancing selection in both comparisons (Extended Data Fig. 3c): the genes encoding Apical membrane antigen 1 (*ama1*) and an uncharacterized conserved protein. Genes under significant selection in both comparisons are enriched for ‘pathogenesis’ and ‘entry into/exit from host cell’ Gene Ontology terms. The increased signatures of selection in the *P. malariae* lineage reflect a genome-wide increase in fixed nonsynonymous mutations in this species (Extended Data Fig. 3d). One of the genes with the highest ratio of nonsynonymous to synonymous mutations in the *P. malariae* and *P. malariae*-like comparison is reticulocyte-binding protein 1a (*RBP1a),* prompting us to hypothesize that human genes encoding transmembrane proteins that act as potential *RBP1a* receptors would have to be conserved in red blood cells between humans and New World primates but not with chimpanzees (Supplementary Information; Methods; Extended Data Table 4). As expected from the recent divergence of *P. malariae* from *P. malariae*-like, more genes were found with signatures of selection in *P. malariae* than between the two *P. ovale* species (Table 1; Extended Data Fig. 3a; Supplementary Information).

Using the added accuracy of manually curated gene sets for both *P. malariae* and *P. o. curtisi* (Table 1; Methods), maximum likelihood trees were constructed using 1,000 conserved single-copy core genes that are present in 12 selected *Plasmodium* species (Fig. 2; Supplementary Information). There is strong evidence that *P. malariae* forms an out-group both to rodent-infective species and to a primate-infective clade that includes *P. vivax.* This phylogenetic arrangement is identical to that found using apicoplast data^6^ but contrasts with other previous studies^1^,^7^,^11^ (Supplementary Information). However, our phylogenetic analysis is based on the most comprehensive amino acid alignment to date, which is enriched for neutrally evolving sites through stringent filtering and has been subjected to a number of different sensitivity tests (Supplementary Information). Assuming consistent mutation rates and generation times across the branches (Supplementary Information), we find that the relative split between the two *P. ovale* species is about five times earlier than the split between *P. falciparum* and *P. reichenowi,* whereas *P. malariae* and *P. malariae*-like seem to have split at a similar time to the latter two (Fig. 2).

Manual curation of about 5,000 gene models of both *P. malariae* and *P. o. curtisi* enabled a detailed exploration of lineage-specific differences in gene content (Table 1), with some paralogous expansions being particularly notable (Extended Data Fig. 1c, d). Genes potentially involved in formation of hypnozoites in *P. ovale—* the lifecycle stage responsible for relapse infections—were also identified (Supplementary Information). The manual curation enabled pseudogenes that are differentially distributed between the two genomes and other human-infective *Plasmodium* species (Extended Data Table 5) to be analysed in ways not possible using computer-annotated draft genome data^7^. For instance, pseudogenes are found among a paralogously expanded family of invasion-associated RBPs (Extended Data Fig. 4a–e), and a homologue of a *P. falciparum* cyclin (PF3D7_1227500) is only pseudogenized in *P. o. wallikeri* and may be linked to the difference in relapse times between the two *P. ovale* species^12^.

Large multigene families are a defining feature that distinguish the genomes of malaria species but are refractory to detailed analysis in non-curated draft genome data. In *P. malariae* and *P. ovale,* approximately 40% of the total genome is subtelomeric. However, the gene content of the subtelomeres differs substantially between the two species (Fig. 3a; Table 1). The breadth and sequence types of the *pir* gene repertoires of the *P. ovale* species are similar to *P. vivax,* whereas *P. malariae* contains only a restricted subset (Extended Data Fig. 5a). The ancient divergence of the two *P. ovale* species is supported by their *pir* repertoires being readily distinguishable (Extended Data Fig. 5b). Despite being sister taxa to *P. ovale,* the *pir* repertoire of rodent malaria parasites, however, appears to be completely different (Extended Data Fig. 5a). Moreover, almost 50% of *pir* genes in *P. malariae* are pseudogenes (compared to 25% in *P. o. curtisi* and 9% in *P. vivax),* suggesting an even smaller functional repertoire.

The most notable difference in the subtelomeres of *P. malariae* is the presence of two large gene families that were not apparent in earlier partial genome data^7^, and which we have termed *fam-l* and *fam-m* (Fig. 3a). Proteins encoded by *fam-l* and *fam-m* show characteristics of proteins that are probably exported from the parasite to the infected red blood cell surface (PEXEL export signal, signal peptide, transmembrane domains, and a variable region). Most *fam-l* and *fam-m* genes face the telomeres and occur as doublets (Fig. 3b), and we found some evidence that they are co-evolving (Extended Data Fig. 5c, d). Proteins encoded by *fam-l* and *fam-m* genes may therefore form heterodimers, a feature not previously seen among subtelomeric gene families in other *Plasmodium* species. Finally, 3D structures of *fam-l* and *fam-m* proteins, predicted with high confidence (template modelling (TM)-score > 0.5)^13^, overlap the crystal structure of the *P. falciparum* RH5 protein (TM-score > 0.8)^14^, with 100% of the RH5 structure covered despite having only 10% sequence similarity (Fig. 3c). RH5 is the only known *P. falciparum* protein that is essential for erythrocyte invasion, through binding to basigin on the erythrocyte surface^15^. The RH5 kite-shaped fold is known to be present in RBP2a in *P. vivax*^16^, and may be a conserved structure necessary for the binding capabilities of all *Rh* and *RBP* genes. This suggests that *fam-l* and *fam-m* genes also have an adhesion role, possibly binding host receptors.

The present study highlights features of host-specific adaptations at several levels: ape to human, primate to rodent and within human hosts. As noted in previous comparative genomics studies involving host switches in the *Plasmodium* genus, invasion-related genes are consistently found to be rapidly evolving. The RBP family is highly expanded (Extended Data Fig. 4a) but its differential distribution across species suggests that RBP3 may be essential for invasion of normocytes (Extended Data Fig. 4c). In contrast to other studies^1^,^7^,^11^, we place the rodent malaria parasites as an outgroup to *P. ovale* but rooted by *P. malariae.* The rodent malaria parasites could therefore more closely model the biology of *P. ovale* than other human-infective species; this indicates that there must have been an ancestral host switch from primates to rodents. The relative dating of speciation events suggests that the move between non-human primates and humans occurred at approximately the same time in two well-separated lineages, suggesting that a common historical event may have promoted host switching and speciation in *Plasmodium* at the time. The much older speciation of the two *P. ovale* parasites and the fact that they have been considered identical until recently shows the limitation of morphology alone in species determination.

Owing to the importance of rapidly evolving multigene families and genome structure, high quality genomes for all human infective species of *Plasmodium* are desperately needed. Although *P. malariae* and *P. ovale* are known to be widespread and common in co-infections with *P. falciparum* (Fig. 1), their low parasitaemia levels have complicated their study and consequently little is known about them. The present study provides a high-quality reference genome for *P. malariae,* thereby providing a step forward in better understanding this elusive species.

## Methods

### Data reporting

No statistical methods were used to predetermine sample size. The experiments were not randomized and the investigators were not blinded to allocation during experiments and outcome assessment.

### Co-infection mining

We aligned the *P. malariae* (AB354570) and *P. ovale* (AB354571) mitochondrial genome sequences against those of *P. falciparum*^18^, *P. vivax*^19^, and *P. knowlesi*^20^ using MUSCLE^21^. For each species, we identified three 15-bp stretches within the *Cox1* gene that contained two or more species-specific single-nucleotide polymorphisms (SNPs). We searched for these 15-bp species-specific barcodes within the sequencing reads of all 2,512 samples from the Pf3K global collection that mapped to the *P. falciparum* mitochondrial genome (http://www.malariagen.net). Samples that contained at least two sequencing reads matching one or more of the 15 bp barcodes for a specific species were considered to be positive for that species (Extended Data Table 2). We found good correspondence between the three different barcodes for each species, with over 80% of positive samples being positive for all three barcodes. We generated pseudo-barcodes by changing two randomly selected nucleotide bases at a time for 10 randomly selected 15 bp region in the *P. vivax*^19^ mitochondrial genome. We did not detect any positive hits using these pseudo-barcodes. As an additional negative control, we searched for *P. knowlesi* co-infections, but did not find any samples positive for this species as expected from the limited geographical range of this species. Two samples (PocGH01, PocGH02) had high numbers of sequencing reads for all three *P. ovale* barcodes and were used for reference genome assembly and SNP calling respectively.

### Parasite material

All *P. ovale* samples were obtained from symptomatic patients diagnosed with a *P. falciparum* infection. The two *P. o. curtisi* samples (PocGH01, PocGH02) identified through co-infection mining (see above), were from two patients that tested positive on a CareStart (HRP2-based) rapid malaria diagnostic test kit at the Navrongo War Memorial hospital, Ghana. One *P. o. wallikeri* sample (PowCR01) and one *P. o. curtisi* sample (PocCR01) were from patients with uncomplicated malaria that tested positive by light microscopy at the Mile 16 Bolifamba Health Centre, Buea, Cameroon. The other *P. o. wallikeri* sample (PowCR02) was obtained from an individual with asymptomatic parasitaemia enrolled through a community survey in Mutengene, Cameroon. For all samples, following consent obtainment, about 2–5 ml of venous blood was obtained and then diluted with one volume of PBS. This was passed through CF11 cellulose powder columns to remove leukocytes before parasite DNA extraction. Ethical approval was obtained for all *P. ovale* samples used in this study.

The two *P. malariae-like* samples, PmlGA01 and PmlGA02, were extracted from Chimpanzee blood (*Pan troglodytes troglodytes,* 9-year-old female and 11-year-old female, respectively) obtained during routine sanitary controls of animals living in a Gabonese sanctuary (Park of La Lékédi, Gabon). Blood collection was performed following international rules for animal health. Within six hours after collection, host white blood cell depletion was performed on fresh blood samples using the CF11 method^22^. After DNA extraction using the Qiagen blood and Tissue Kit and detection of *P. malariae* infections by *Cytb* PCR and sequencing^23^, the samples went through a whole-genome amplification step^24^.

One *P. malariae* sample, PmMA01, was collected from a patient with uncomplicated malaria in Faladje, Mali. Venous blood (2–5 ml) was depleted of leukocytes within 6 h of collection through CF11 cellulose powder columns as previously described^25^. The study protocol was approved by the Ethics Committee of Faculty of Medicine and Odontomatology and Faculty of Pharmacy, Bamako, Mali.

Four samples of *P. malariae* were obtained from travellers returning to Australia with malaria. PmUG01 and PmID01 were sourced from patients returning from Uganda and Papua Indonesia, respectively, who presented at the Royal Darwin Hospital, Darwin, with microscopy-positive *P. malariae* infection. PmMY01 was sourced from a patient presenting at the Queen Elizabeth Hospital, Sabah, Malaysia, with microscopy-positive *P. malariae* infection. Patient sample PmGN01 was collected from a patient who presented to Royal Brisbane and Women’s Hospital in 2013 on return from Guinea. Venous blood samples were subject to leukodepletion within 6 h of collection. PmUG01 was leukodepleted using a commercial Plasmodipur filter (EuroProxima, The Netherlands); custom-made cellulose-based filters were used for PmID01 and PmMY01, whereas PmGN01 was leukodepleted using an inline leukodepletion filter present in the venesection pack (Pall Leukotrap; WBT436CEA). DNA extraction was performed on filtered blood using commercial kits (QIAamp DNA Blood Midi kit, Qiagen). For samples PmUG01, PmID01 and PmMY01, ethical approval for the sample collection was obtained from the Human Research Ethics Committee of NT Department of Health and Families and Menzies School of Health Research (HREC-2010-1396 and HREC-2010-1431) and the Medical Research Ethics Committee, Ministry of Health Malaysia (NMRR-10-754-6684). For sample PmGN02, ethical approval was obtained from the Royal Brisbane and Women’s Hospital Human Research Ethics Committee (HREC/10/QRBW/379) and the Human Research Ethics Committee of the Queensland Institute of Medical Research (p1478).

### Sample preparation and sequencing

One *P. malariae* sample, PmUG01, was selected for long-read sequencing, using Pacific Biosciences (PacBio), owing to its low host contamination and abundant DNA. Passing through a 25-mm blunt-ended needle, 6 (μg of DNA was sheared to 20–25 kb. SMRT bell template libraries were generated using the PacBio issued protocol (20 kb Template Preparation using the BluePippin Size-Selection System). After a greater than 7 kb size-selection using the BluePippin Size-Selection System (Sage Science), the library was sequenced using P6 polymerase and chemistry version 4 (P6/C4) in 20 SMRT cells (Supplementary Information).

The remaining isolates were sequenced with Illumina Standard libraries of 200–300 bp fragments and amplification-free libraries of 400–600 bp fragments were prepared^26^ and sequenced on the Illumina HiSeq 2000 v3 or v4 and the MiSeq v2 according to the manufacturer’s standard protocol (Supplementary Information). Raw sequence data was deposited in the European Nucleotide Archive (Supplementary Information).

### Genome assembly

The PacBio-sequenced *P. malariae* sample, PmUG01, was assembled using HGAP^27^ with an estimated genome size of 100 Mb to account for the host contamination (~85% human). The resulting assembly was corrected initially using Quiver^27^, followed by iCORN^28^. PmUG01 consisted of multiple haplotypes, with the majority haplotype being used for the iCORN^28^, and a coverage analysis was performed to remove duplicate contigs. Additional duplicated contigs were identified using a BLASTN^29^ search, with the shorter contigs being removed if they were fully contained within the longer contigs or merged with the longer contig if their contig ends overlapped. Host contamination was removed by manually filtering on GC, coverage, and BLASTN hits to the non-redundant nucleotide database^29^.

The Illumina-based genome assemblies for *P. o. curtisi, P. o. wallikeri,* and *P. malariae-like* were performed using MaSURCA^30^ for samples PocGH01, PowCR01, and PmlGA01 respectively. To confirm that the assemblies were indeed *P. ovale,* we mapped existing *P. ovale* capillary reads to the assemblies (http://www.ncbi.nlm.nih.gov/Traces/trace.cgi?view=search, using as search terms: CENTER_PROJECT = “PO” and CENTER_NAME = “SC”). Before applying MaSURCA^30^, the samples were mapped to the *P. falciparum* 3D7 reference genome^18^ to remove contaminating reads. The draft assemblies were further improved by iterative uses of SSPACE^31^, GapFiller^32^ and IMAGE^33^. The resulting scaffolds were ordered using ABACAS^34^ against the *P. vivax* PVP01 (ref. 35) (http://www.genedb.org/Homepage/PvivaxP01) assembly (both *P. ovale*) or against the *P. malariae* PacBio assembly (*P. malariae-like).* The assemblies were manually filtered on GC, coverage, and BLASTN hits to the non-redundant nucleotide database^29^. iCORN^28^ was used to correct frameshifts. Finally, contigs shorter than 1 kb were removed.

Using two more samples, PocGH02 and PowCR02, additional draft assemblies of both *P. ovale* species were produced using MaSURCA^30^ followed by RATT^36^ to transfer the gene models from the high-quality assemblies.

The genome sequences and annotations are currently available on GeneDB for *P. malariae* (http://www.genedb.org/Homepage/Pmalariae) and for *P. ovale curtisi* (http://www.genedb.org/Homepage/Povale). The genome sequences have been deposited into the European Nucleotide Archive (http://www.ebi.ac.uk/ena). Accession numbers for all reads generated for this study can be found in Supplementary Information. Accession identifiers for the assembled genome sequences can be found in Supplementary Information.

### Gene annotation

RATT^36^ was used to transfer gene models on the basis of synteny conserved with other sequenced *Plasmodium* species (*P. falciparum*^18^, *P. vivax*^19^, *P. berghei*^37^, and *P. gallinaceum* (http://www.genedb.org/Homepage/Pgallinaceum). In addition, genes were predicted *ab initio* using AUGUSTUS^38^, trained on a set consisting of manually curated *P. malariae* and *P. ovale* genes respectively. Non-coding RNAs and tRNAs were identified using Rfam 12.0 (ref. 39). Gene models were then manually curated for both the *P. malariae* and *P. o. curtisi* reference genomes, using Artemis^40^ and the Artemis Comparison Tool^41^. These tools were also used to manually identify deleted and disrupted genes (Extended Data Table 5). The *P. malariae-*like and *P. o. wallikeri* genomes were both annotated using the Companion tool^42^.

### Phylogenetics

**Figure 2:**
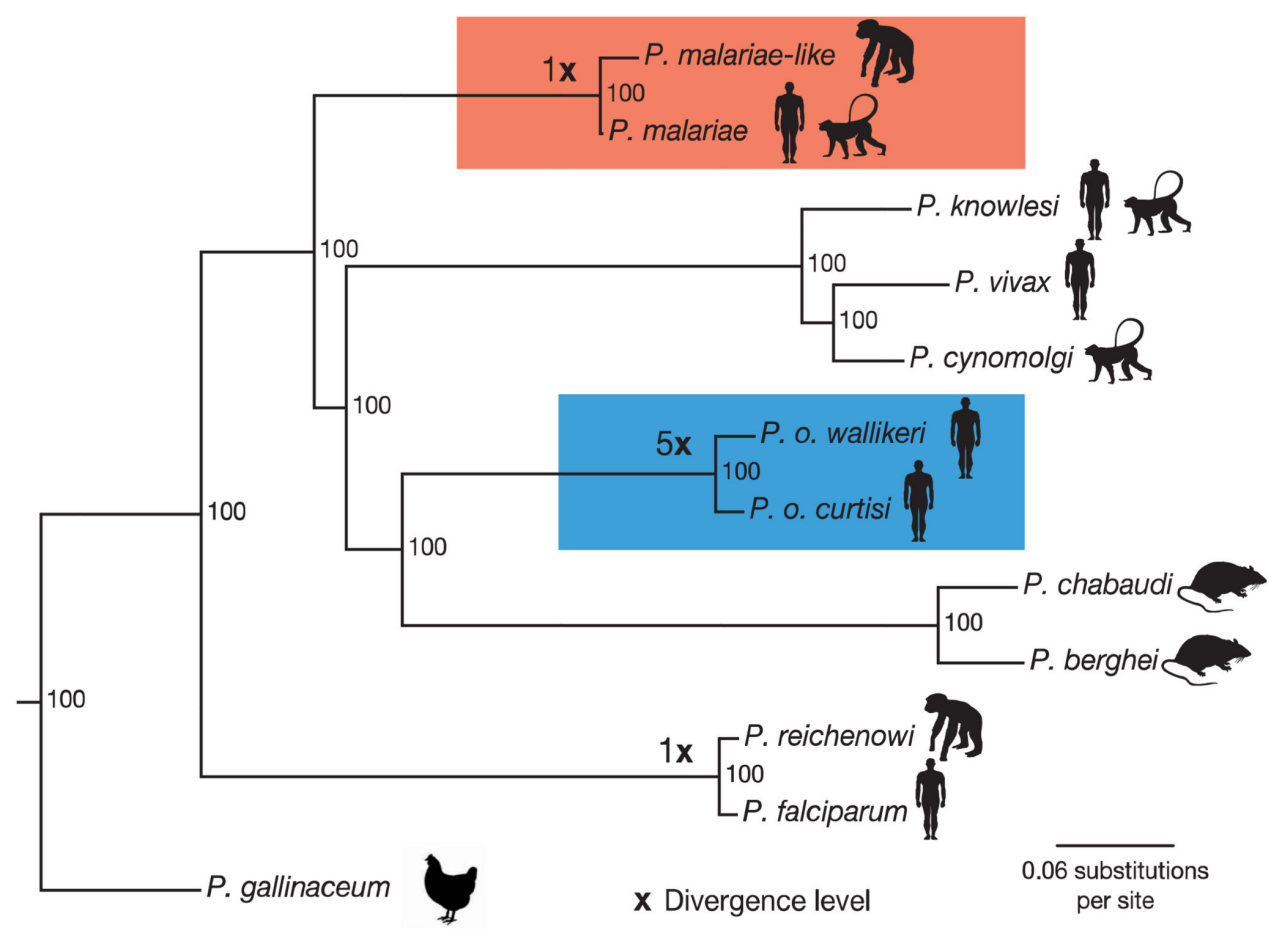
Phylogenetic tree of the *Plasmodium* genus. Maximum likelihood phylogenetic tree of the *Plasmodium* genus, showing the *P. malariae* clade (red) and the *P. ovale* clade (blue) together with the divergence levels of the species as calibrated to the *P. falciparum* and *P. reichenowi* split (×). Using a previously published date of 3.0–5.5 million year ago for the *P. falciparum* and *P. reichenowi* split^17^, we thereby date the *P. ovale* split to 20.3 million years ago and the *P. malariae* split to 3.5 million years ago. Silhouettes show host specificity of the different species. Values at branching points are bootstrap values (Methods).

Following orthologue assignment using BLASTP^29^ and OrthoMCL^43^, amino acid sequences of 1,000 core genes from 12 *Plasmodium* species (*P. gallinaceum*^44^, *P. falciparum*^18^, *P. reichenowi*^10^, *P. knowlesi*^20^, *P. vivax* P01 (ref. 35), *P. cynomolgi*^45^, *P. chabaudi*^37^, *P. berghei*^37^, and the four assemblies produced in this study) were aligned using MUSCLE^21^. The alignments were cleaned using GBlocks^46^ with default parameters to remove non-informative and gapped sites. The cleaned non-zero length alignments were then concatenated. This resulted in an alignment of 421,988 amino acid sites per species. The optimal substitution model for each gene partition was determined by running RAxML^47^ for each gene separately using all implemented substitution models. The substitution models with the minimum Akaike Information Criterion on a guide maximum parsimony tree were used for each gene partition. A maximum likelihood phylogenetic tree was constructed using RAxML^47^ version 8.2.4. with 100 bootstraps^48^ (Fig. 2). To confirm this tree, we used different phylogenetic tools including PhyloBayes^49^ and PhyML^50^, a number of different substitution models within RAxML, starting the tree search from the commonly accepted phylogenetic tree, and removing sites in the alignment which supported significantly different trees (Supplementary Information). Figtree was used to display and colour the tree (http://tree.bio.ed.ac.uk/software/figtree/).

A phylogenetic tree of four *P. malariae* (PmID01, PmGN01, PmGN02, PmMY01) and all *P. malariae-*like samples (PmlGA01, PmlGA02) was generated using PhyML^50^ on the basis of all *P. malariae* genes. For each sample, the raw SNPs, as called using the SNP calling pipeline (see below), were mapped onto all genes to morph them into sample-specific gene copies using BCFtools^51^. Amino acid alignments for all genes were concatenated and cleaned using GBlocks^46^ with default parameters.

### Divergence dating

Species divergence times were estimated using the Bayesian inference tool G-PhoCS^52^, a software which uses unlinked neutrally evolving loci and a given phylogeny to estimate demographic parameters. One additional sample per assembly (PmGN01 for *P. malariae,* PocGH02 for *P. o. curtisi,* PowCR02 for *P. o. wallikeri,* and PmlGA02 for *P. malariae-*like) was used to morph the respective assembly using iCORN^28^. Regions in the genomes without mapping were masked, as iCORN^28^ would not have morphed them. Unassigned contigs and subtelomeric regions were removed for this analysis owing to the difficulty of alignment. Repetitive regions in the chromosomes of the four assemblies and the four morphed samples were masked using Dustmasker^53^ and then the chromosomes were aligned using FSA^54^. The *P. o. wallikeri* and the *P. o. curtisi* chromosomes were aligned against each other, as were the *P. malariae* and *P. malariae-*like chromosomes. The alignments were split into 1 kb loci, removing those that contained gaps, masked regions, and coding regions to conform with the neutral loci prediction of G-PhoCS^52^. G-PhoCS^52^ was run for one million Markov Chain Monte Carlo (MCMC)-iterations with a sample-skip of 1,000 and a burn-in of 10,000 for each of the two-species pairs. Follow-up analyses using Tracer (http://beast.bio.ed.ac.uk/Tracer) confirmed that this was sufficient for convergence of the MCMC chain in all cases. In the model, we assumed a variable mutation rate across loci and allowed for on-going gene flow between the populations. The tau values obtained from this were 0.0049 for *P. malariae* and 0.0434 for *P. ovale*.

The tau values were used to calculate the date of the split, using the formula (tau × *G*) / *m*, where *G* is the generation time in years and *m* is the mutation rate. Testing a number of different generation time and mutation rate estimates in order to optimize the *P. falciparum* and *P. reichenowi* split to 4 million years ago as estimated previously^17^, we found a mutation rate of 3.8 × 10^−10^ SNPs per site per lifecycle^55^ and a generation time of 65 days^56^ to generate this previously published date^17^. For *P. malariae,* a generation time of 100 days was used owing to the longer intra-erythrocytic cycle.

### 3D structure prediction

The I-TASSER^57^ version 4.4 online web server^58^ (http://www.zhanglab.ccmb.med.umich.edu/I-TASSER) was used for 3D protein structure prediction. Predicted structures with a TM-score of over 0.5 were considered reliable as suggested in the I-TASSER user guidelines^13^. TM-align^14^, as implemented in I-TASSER^58^, was used to overlay the predicted protein structure with existing published protein structures.

### Hypnozoite gene search

Using the OrthoMCL^43^ clustering between all sequenced *Plasmodium* species used for the phylogenetic analysis (see above), we examined clusters containing only genes of hypnozoite-forming species: *P. vivax* P01 genes, *P. cynomolgi*^45^ genes and genes of both of the *P. ovale* species.

Additionally, we examined *P. o. curtisi* orthologues of previously published hypnozoite gene candidates^45^, looking in the 1 kb 5′ upstream region for any of the four ApiAP2 motifs^59^ involved in sporozoite regulation and expression: GCATGC (PF3D7_1466400), GCCCCG (PF3D7_1342900), TAAGCC (PF3D7_1342900), and TGTTAC (PF3D7_0420300) (Supplementary Information).

### Gene family analysis

All *P. malariae, P. ovale,* and *P. vivax* P01 genes were compared pairwise using BLASTP^29^, with genes having a minimum local BLAST hit of 50% identity over 150 amino acids or more being considered connected. These gene connections were visualized in Gephi^60^ using a Fruchterman-Reingold^61^ layout and with unconnected genes removed.

*P. malariae, P. o. curtisi* and *P. o. wallikeri* protein sequences for *Plasmodium* interspersed repeat (*pir*) genes, excluding pseudogenes, were combined with those from *P. vivax* P01 (ref. 35), *P. knowlesi*^20^, *P. chabaudi* AS v3 (http://www.genedb.org/Homepage/Pchabaudi), *P. yoelii yoelii 17X* v2 (ref. 37), and *P. berghei* v3 (ref. 62). Sequences were clustered using tribeMCL^63^ with blast *e*-value 0.01 and inflation 2. This resulted in 152 subfamilies. We then excluded clusters with one member. The number of genes per species in each subfamily were plotted in a heatmap using the heatmap.2 function in ggplots in R-3.1.2.

The *pir* genes from two *P. o. curtisi* and two *P. o. wallikeri* assemblies (two high-quality and two draft genome assemblies) were compared pairwise using BLASTP^29^ with a 99% identity over a minimum of 150 amino acids cutoff. The gene–gene connections were visualized in Gephi^60^ using a Fruchterman-Reingold^61^ layout after removing unconnected genes.

### Mirror tree analysis

Using Artemis^40^, 79 *fam-m* and *fam-l* doublets that were confidently predicted as being paired-up were manually selected on the basis of their dispersal throughout the subtelomeres of different chromosomes. The Mirrortree^64^ web server (http://csbg.cnb.csic.es/mtserver/) was used to construct mirror trees for these 79 doublets. 35 doublets with recent branching from another doublet were manually selected to enrich for genes under recent selection (Extended Data Fig. 5c). To control for chance signals of co-evolution on the basis of their subtelomeric location, the same methodology was repeated by choosing 79 *pir* genes in close proximity of *fam-m* genes as ‘pseudo-doublets’ for analysis and paired up in the Mirrortree^64^ web server (Extended Data Fig. 5d).

### Reticulocyte-binding protein (RBP) phylogenetic plot

Full-length RBP genes were manually inspected using ACT^41^ and verified to either be functional or pseudogenized by identification of sequencing reads in other samples that confirm mutations inducing premature stop codons or frameshifts. All functional RBPs were aligned using MUSCLE^21^ and cleaned using GBlocks^46^ with default parameters. PhyML^50^ was used to construct a phylogenetic tree of the different RBPs (Extended Data Fig. 4a). Figtree was used to colour the tree (http://tree.bio.ed.ac.uk/software/figtree/).

### SNP calling

Additional *P. malariae* (PmMY01, PmID01, PmMA01, PmGN01) and *P. o. curtisi* (PocGH01, PocGH02, PocCR01) samples were mapped back against the reference genomes using SMALT (-y 0.8, -i 300) (Supplementary Information). As outgroups, *P. malariae-like* (PmlGA01, PmlGA02) and *P. o. wallikeri* (PowCR01, PowCR02) were also mapped against the *P. malariae* and *P. o. curtisi* genomes respectively. The resulting ‘bam’ formatted files were merged for either of the two genomes, and GATK^65^ Unified Genotyper was used to call SNPs from the merged bam files (Supplementary Information). As per GATK^65^ best practices, SNPs were filtered by quality of depth (QD > 2), depth of coverage (DP > 10), mapping quality (MQ > 20), and strand bias (FS < 60). Additionally, all sites for which we had missing data for any of the samples or for which we had heterozygous calls were filtered. Finally, we filtered sites that were masked using Dustmasker^53^ to remove repetitive and difficult to map regions. The same methodology was also applied to two *P. vivax* samples (SRR3400910 and SRR332566) and two *P. falciparum* Pf3K field samples (PF0066-C and PF0038-C, see https://www.malariagen.net/projects/pf3k) for comparative purposes.

### Molecular evolution analysis

To calculate the nucleotide diversity for the different species, we extracted all filtered SNPs in the genomes excluding the subtelomeres. We then counted the number of pairwise differences between the different samples divided by the resulting genome size, comprising three comparisons for species with three samples (*P. malariae, P. o. curtisi, P. vivax, P. falciparum*) and one comparison for species with two samples (*P. o. wallikeri*, *P. malariae*-like). These estimates were then averaged by species (Table 1).

The filtered SNPs were used to morph the reference genomes using BCFtools^51^ for each sample, from which sample-specific gene models were obtained. Nucleotide alignments of each gene were then generated. Codons with alignment positions that were masked using Dustmasker^53^ were excluded. For each alignment (that is, gene), we calculated Hudson–Kreitman–Aguadé (HKA)^66^, McDonald–Kreitman (MK)^67^, and *K*_a_/*K*_s_^68^ values (see below). Subtelomeric gene families and pseudogenes were excluded from the analysis. The results were analysed and plotted in RStudio (http://www.rstudio.com/).

For the HKA^66^, we counted the proportion of pairwise nucleotide differences intra-specifically (that is, within *P. malariae* and within *P. o. curtisi*) and inter-specifically (that is, between *P. malariae* and *P. malariae-like,* between *P. o. curtisi* and *P. o. wallikeri).* The intraspecific comparisons were averaged to obtain the nucleotide diversity π of the genes and these were divided by the average inter-specific comparisons, the nucleotide divergence, to get the HKA ratio (HKAr) for each gene.

The MK test^67^ was performed for each gene by obtaining the number of fixed and polymorphic changes, as well as a *P* value, as previously described^69^ and then calculating the skew as log_2_(((*N*_poly_ + 1) / (*S*_poly_ + 1)) / ((*N*_fix_ + 1) / (*S*_fix_ + 1))), where *N*_poly_ and *N*_fix_ are polymorphic and fixed non-synonymous substitutions respectively, and *S*_poly_ and *S*_fix_ refer to the synonymous substitutions.

To calculate the average *K*_a_/*K*_s_ ratio^68^, we took the cleaned alignments of the MK test, extracting the pairwise sequences of *P. malariae* and *P. malariae*-like (and of *P. o. curtisi* and *P. o. wallikeri).* The ‘Bio::Align::DNAStatistics’ module was used to calculate the *K*_a_/*K*_s_ values for each pair^70^, averaging across samples within a species.

Using existing RNA sequencing data from seven different life-cycle stages in *P. falciparum*^71^, reads were mapped against spliced gene sequences (exons, but not UTRs) from the *P. falciparum* 3D7 reference genome^18^ using Bowtie2 (ref. 72) v2.1.0 (-a -X 800 –x). Read counts per transcript were estimated using eXpress v1.3.0 (ref. 73). Genes with an effective length cutoff below 10 in any sample were removed. Summing over transcripts generated read counts per gene. Numbers were averaged for all gametocyte stages and for all blood stages. Genes with no stage having 10 or more reads were classified as being expressed elsewhere. Genes in *P. malariae* and *P. ovale* were classified by the maximum expression stage of their *P. falciparum* orthologue if the difference between the maximum expression stage and the second highest stage was larger than the difference between the second and third highest stage, otherwise the gene was classified as having no peak expression.

The Gene Ontology term enrichment analysis was performed in R, using TopGO^74^. As a Gene Ontology database, the predicted Gene Ontology terms from the *P. falciparum* 3D7 genes orthologous to the *P. malariae* and *P. o. curtisi* genes included in the analysis were used. Collated tables for all molecular evolution measures for all genes can be found in Source Data File 1 for *P. malariae* and *P. o. curtisi*.

### RBP1a receptor search

To find the putative RBP1a receptor, we performed an OrthoMCL^43^ clustering between human, chimpanzee^75^, and common marmoset^76^ genes. *P. brasilianum (P. malariae*) results in chronic infection in the common marmoset^77^. Genes without transmembrane domains as well as those annotated as ‘predicted’ were removed. To limit false positives, all remaining genes were searched against the chimpanzee genes using BLASTP^29^ with a threshold of 1 × 10^−10^.

### Data deposition statement

All raw data has been deposited as described in Supplementary Information. Assembled genome sequences can be found under the study PRJEB14392 (http://www.ebi.ac.uk/ena/data/view/PRJEB14392). The individual accession numbers are as follows for PmUG01 (contig accession: FLRL01000001–FLRL01000047; chromosome accession: LT594622–LT594637), PocGH01 (contig accession: FLRI01000001–FLRI01000638; chromosome accession: LT594582–LT594597), PowCR01 (contig accession: FLRJ01000001–FLRJ01000771; chromosome accession: LT594505-LT594520) and PmlGA01 (contig accession: FLRK01000001–FLRK01000035; chromosome accession: LT594489–LT594503).

## Extended Data

**Extended Data Figure 1:**
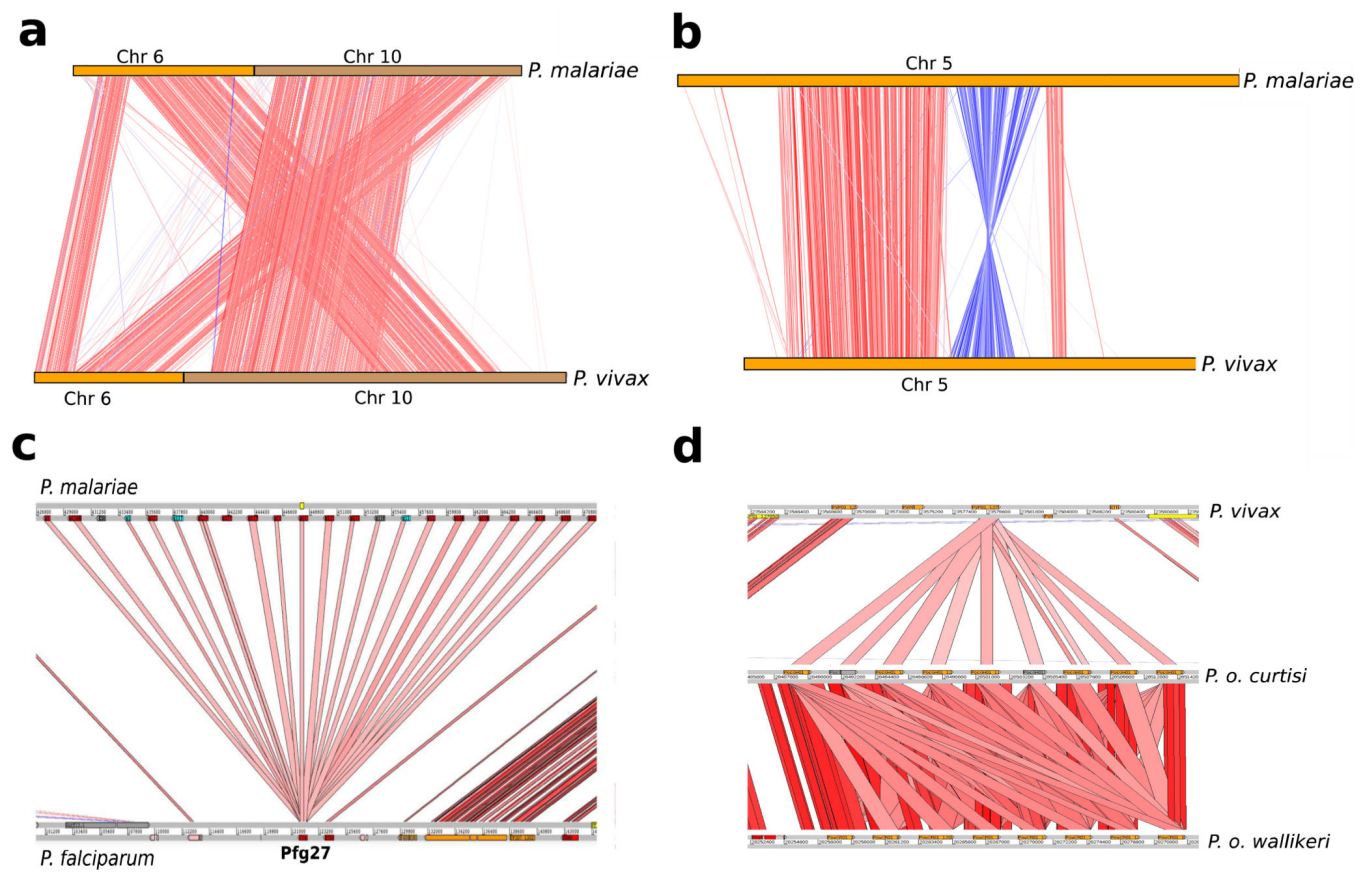
Large genomic changes in the *P. malariae* and *P. ovale* genome sequences. **a,** Artemis Comparison Tool (ACT)^41^ view showing reciprocal translocation of chromosomes 6 and 10 in *P. malariae.* The red lines indicate blast similarities, chromosome 6 in orange and chromosome 10 in brown. **b,** ACT^41^ view showing a pericentric inversion in chromosome 5 of *P. malariae.* Red lines indicate BLAST similarities and blue lines indicate inverted BLAST hits. **c,** Expansion of 22 copies (20 functional) of Pfg27 in *P. malariae* (top) compared to a single copy in *P. falciparum* (bottom) with red lines indicating BLAST similarities. Functional genes are in red and pseudogenes in grey. This compares to only 17 Pfg27 copies described previously^7^, which were found on six separate contigs, while all Pfg27 copies described here are found on the same contig. **d,** Expansion of PVP01_1270800 (PF3D7_1475900 in *P. falciparum),* a gene with no known function, in *P. o. curtisi* and *P. o. wallikeri,* with different copy numbers in each, compared to the one copy in *P. vivax.* Functional genes shown in orange and pseudogenes shown in grey. This gene family was recently named KELT^7^, and we confirm the 8 copies present in *P. o. wallikeri,* but show that *P. o. curtisi* has 9 copies, two of which are pseudogenes.

**Extended Data Figure 2:**
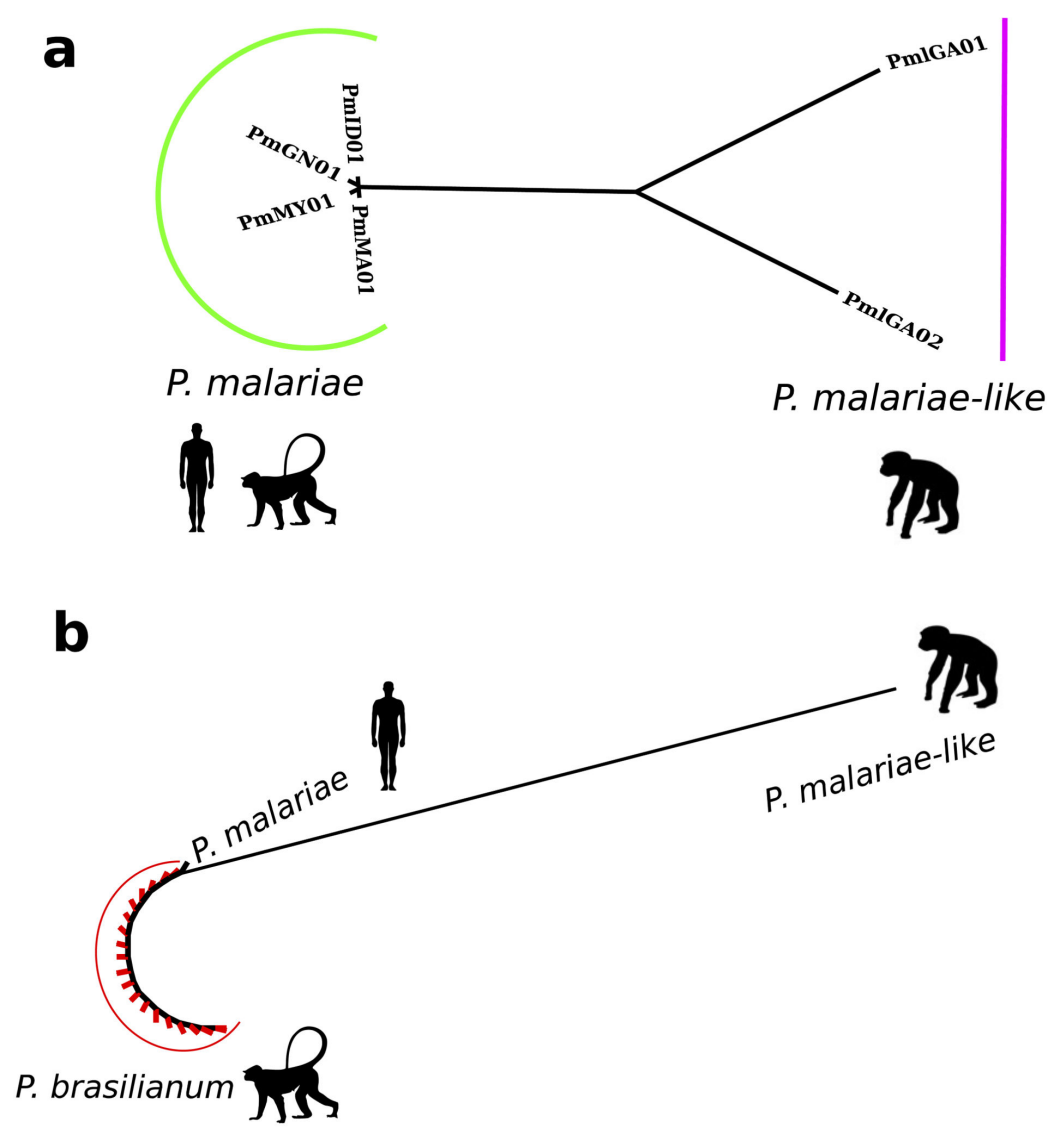
*P. malariae-like* has significantly longer branch lengths than *P. malariae,* and *P. brasilianum* is identical to *P. malariae.* **a,** A phylogenetic tree of all *P. malariae* and *P. malariae-*like samples generated using PhyML^50^ on the basis of all *P. malariae* genes. *P. malariae* samples are indicated by a green bar and *P. malariae--like* samples are indicated by a purple bar. Silhouettes represent host infectivity. **b,** A PhyML^50^ phylogenetic tree of all *P. brasilianum* 18S rRNA sequences^9^, indicated by a red bar and red tip branches, and the corresponding 18S rRNA sequences from the *P. malariae* and *P. malariae*-like assemblies, labelled as such. Silhouettes represent the host origin for each sample.

**Extended Data Figure 3:**
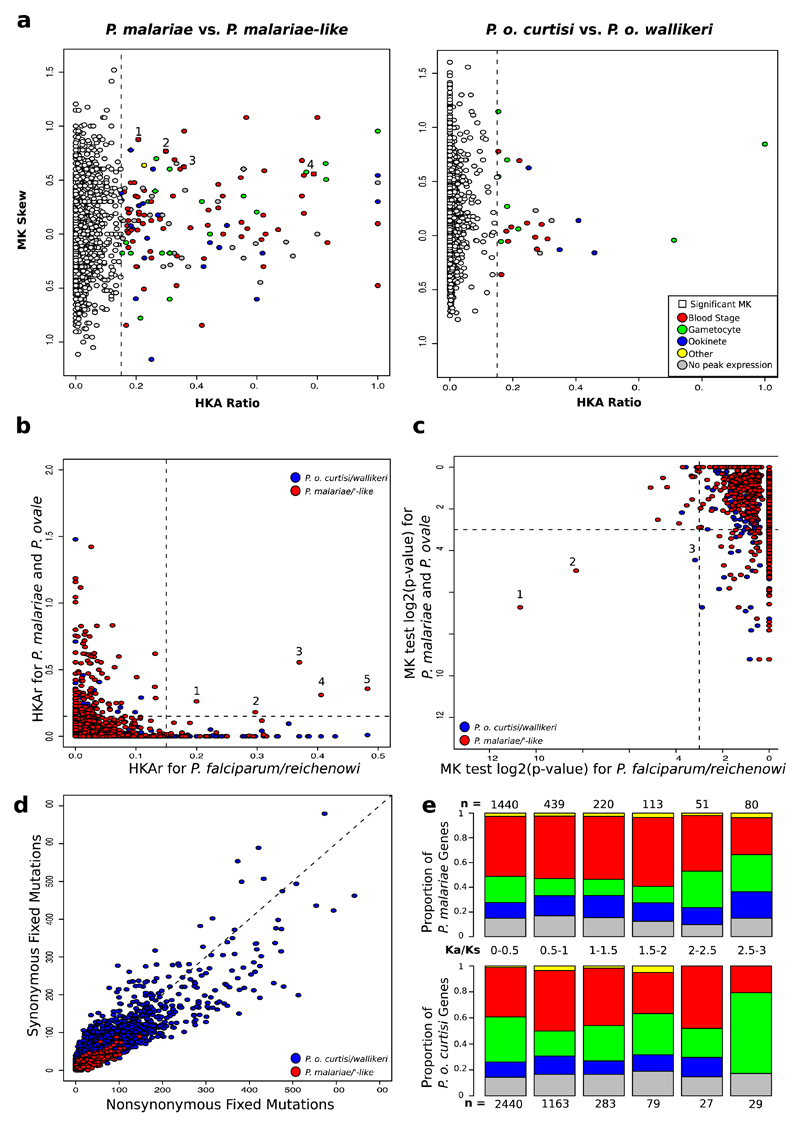
Different population genetics in *P. malariae* and *P. o. curtisi.* **a,** HKA ratio and MK Skew for both *P. malariae* versus *P. malariae-*like (left) and *P. o. curtisi* versus *P. o. wallikeri* (right). Genes with high HKAr values (>0.15, vertical line) are coloured by the peak expression of their orthologue in *P. falciparum*^71^ (red, blood stage; green, gametocyte; blue, ookinete; yellow, other; grey, no peak expression) (Methods). Genes with high HKAr and a significant MK skew (square symbols): (1) merozoite surface protein 9, PF3D7_1228600; (2) rRNA (adenosine-2'-O-)-methyltransferase, PF3D7_1429400; (3) merozoite surface protein 1, PF3D7_0930300; (4) formin 1, PF3D7_0530900. **b,** Gene-wide HKAr values for the *P. falciparum* to *P. reichenowi* comparison, described earlier^10^, versus HKAr for the *P. o. curtisi* to *P. o. wallikeri* (blue) and the *P. malariae* to *P. malariae*-like (red) comparisons. Five genes show significant HKAr (> 0.15) values for both comparisons: (1) ferrodoxin reductase-like protein (PF3D7_0720400); (2) EGF-like membrane protein (PF3D7_0623300); (3) ADP/ATP carrier protein (PF3D7_1004800); (4) merozoite surface protein 1 (PF3D7_0930300); (5) conserved *Plasmodium* protein (PF3D7_0311000). **c,** log2 of *P* values of gene-wide MK tests for the *P. falciparum* to *P. reichenowi* comparison10 by *P. o. curtisi* to *P. o. wallikeri* (blue) and *P. malariae* to *P. malariae-like* (red) comparisons. Three genes have significant MK skews (log2(P) < −3) for both comparisons: (1) glideosome-associated connectyor (PF3D7_1361800); (2) apical membrane antigen 1 (PF3D7_1133400); (3) NAD(P)H-dependent glutamate synthase (PF3D7_1435300). **d,** Nonsynonymous versus synonymous fixed mutations per gene for both the *P. o. curtisi* to *P. o. wallikeri* (blue) and the *P. malariae* to *P. malariae-*like (red) comparisons. Whereas the former has most genes centred around the x = y line, the latter has most genes below this line with more nonsynonymous than synonymous mutations, indicative of an ancestral bottleneck. **e,** Bar plot of proportion of *P. malariae* (above) and *P. o. curtisi* (below) genes expressed at different stages (no peak expression (grey), ookinete (blue), gametocyte (green), intraerythrocytic (red), and other stage (yellow)) binned by *K_a_/K_s_*, with the number of genes in each bin displayed (n). *P. o. curtisi* genes with very high *K_a_/K_s_* values (> 2.5) are enriched for genes with peak expression in gametocyte.

**Extended Data Figure 4:**
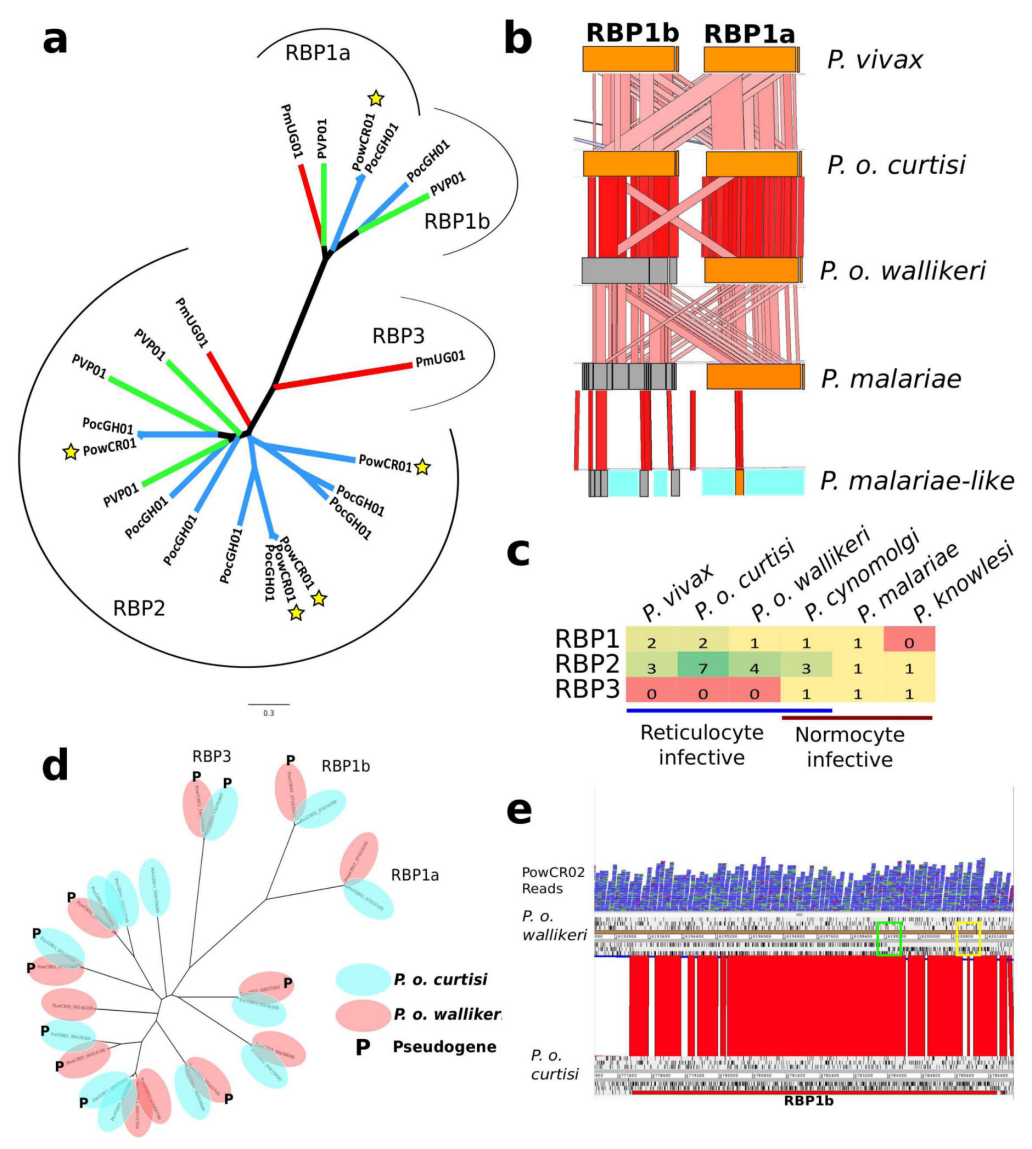
Reticulocyte-binding protein changes in *P. malariae* and *P. ovale.* **a,** Phylogenetic tree of all full-length functional RBPs in *P. malariae* (red branches), *P. o. curtisi* (blue branches without stars), *P. o. wallikeri* (blue branches with stars), and *P. vivax* (green branches). Brackets indicate the different subclasses of RBPs: RBP1a, RBP1b, RBP2 and RBP3. **b,** ACT^41^ view of functional (orange) and pseudogenized (grey) RBP1a and RBP1b in five species (*P. vivax, P. o. curtisi, P. o. wallikeri, P. malariae* and *P. malariae*-like). Blue indicates assembly gaps. Red bars between species indicate level of sequence similarity, with darker colour indicating higher similarity. **c,** Number of RBP genes in each of the three RBP classes (RBP1, RBP2, RBP3) by species (*P. vivax, P. o. curtisi, P. o. wallikeri, P. cynomolgi, P. malariae, P. knowlesi*) grouped by erythrocyte invasion preference (reticulocyte versus normocyte). **d,** PhyML^50^ generated phylogenetic tree of all RBP genes over 1 kb long in *P. o. curtisi* and *P. o. wallikeri.* Pseudogenes are denoted with **(P).** Multiple functional RBP2 genes match up with pseudogenized copies in the other genome. **e,** ACT^41^ view of RBP1b in red for *P. o. curtisi* (bottom) and the corresponding disrupted open reading frame in *P. o. wallikeri* (top), with black ticks indicating stop codons. Reads (in blue) from an additional *P. o. wallikeri* sample (PowCR02) confirm the bases introducing the frameshift (green box) and premature stop codon (yellow box) in RBP1b.

**Extended Data Figure 5:**
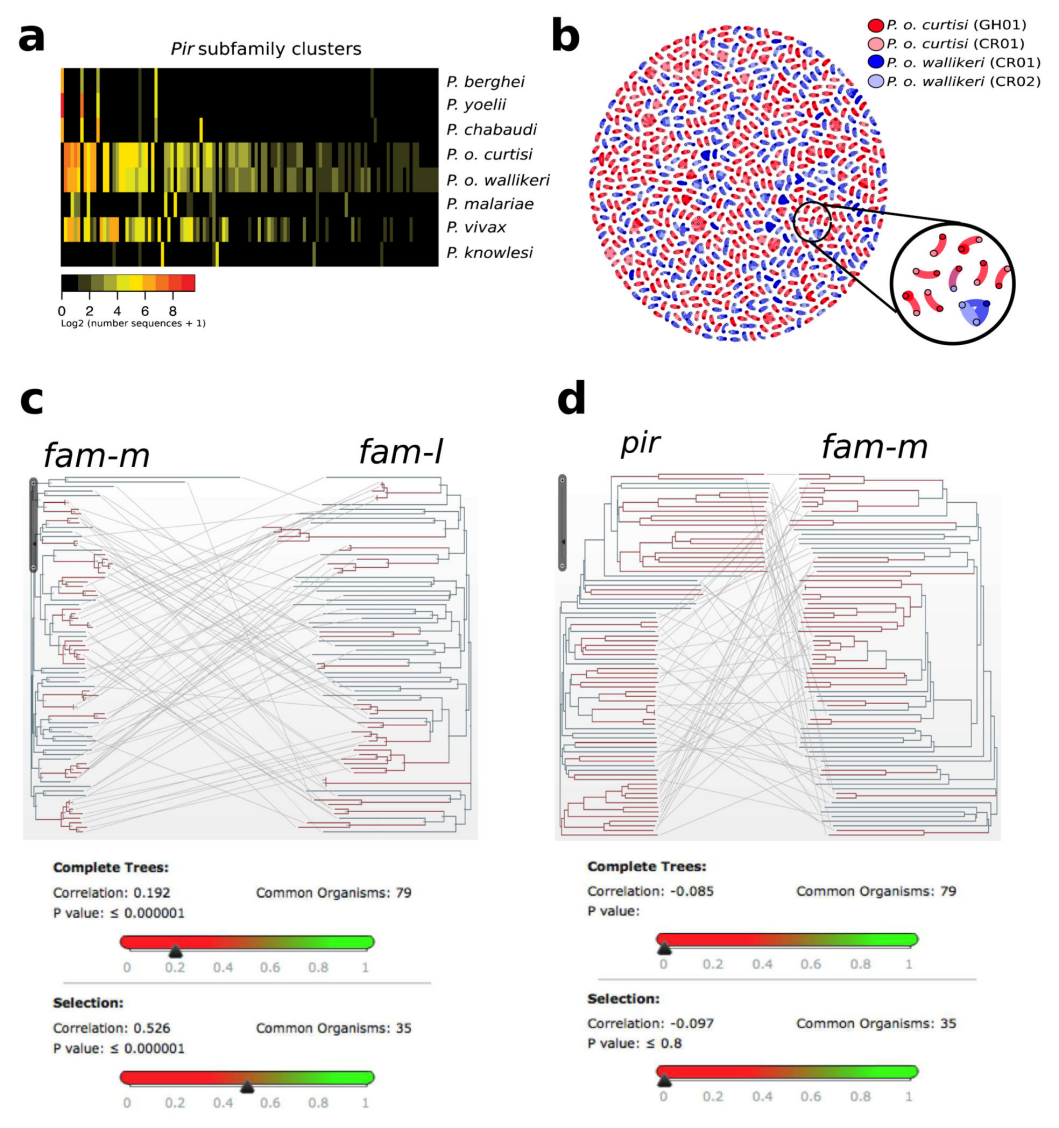
Subtelomeric gene family dynamics in *P. ovale* and *P. malariae*. **a,** Heat map showing the sharing of *pir* subfamilies between different species based on tribeMCL^63^. Columns show *pir* subfamilies and rows show species. Colours indicate the number of genes classified into each subfamily for each species. Subfamilies were ordered by size, species were ordered for clarity. *pir* genes in rodent-infective species fall into a small number of well-defined families. Those in *P. vivax, P. malariae* and *P. ovale,* however, are much more diverse. There is little overlap between rodent subfamilies and human-infective subfamilies, despite *P. ovale* being a sister taxa to the rodent-infecting species. *P. knowlesi* has some sharing with other species, but its largest families are species specific, suggesting it has undergone specialization of its *pir* repertoire. **b,** Gene network of *pir* genes for both high-quality assemblies of *P. o. curtisi* (dark red) and *P. o. wallikeri* (dark blue) and draft assemblies of each (light red and light blue respectively). *pir* genes with BLASTP^29^ identity hits of 99% and over 150 amino acids become connected in the graph. Genes without connections were removed. There is one connection between the two species (circled in black and with a zoomed in version), 801 between the *P. o. curtisi* assemblies, 524 between the *P. o. wallikeri* assemblies, 527 on average within each *P. o. curtisi* assembly, and 423 on average within each *P. o. wallikeri* assembly. This indicates that there is considerably less sharing of *pir* genes between the two *P. ovale* species than within each species, as expected if the two do not recombine with each other. **c,** Mirror tree^64^ for 79 *fam-m* and *fam-l* doublets, where the two phylogenetic trees correspond to either of the families with lines connecting branch tips of the same doublet. 35 branches (red) were manually selected owing to exhibiting recent branching. Inset below shows the correlations as calculated by the Mirrortree webserver^64^ between the two trees for all branches (above, correlation = 0.19, *P* < 0.001) and red branches (below, correlation = 0.53, *P* < 0.001). This shows that the two families are co-evolving, especially when doublets that recently branched are selected, suggesting that the co-evolution may be disrupted over longer periods of time, potentially through recombination. **d,** Mirror tree^64^ for 79 *pir* and *fam-m* pseudo-doublets (Methods), where the two phylogenetic trees correspond to either of the families with lines connecting branch tips of the same doublet. We manually selected 35 branches (red) as they exhibited recent branching. Inset shows the correlations as calculated by the Mirrortree webserver^64^ between the two trees for all branches (above, correlation = −0.09, *P* > 0.05) and red branches (below, correlation = −0.10, *P* > 0.05). This shows that the two families are not co-evolving, and that subtelomeric location does not produce sporadic signals of co-evolution.

**Extended Data Table 1:** Assembly and annotation statistics for the recently described assemblies compared to the present assemblies

	PmUG01	Pmal	PmlGA01	PocGH01	Poc1	Poc2	PowCR01	Pow1	Pow2
Size (Kb)	33,618	31,925	23,693	33,485	34,519	38,010	33,579	35,285	35,192
Largest (kb)	3,564	56	3,177	2,946	94	491	3,061	569	657
Average (kb)	534	4	474	22	9	17	43	26	22
Gaps	0	2,236	3,697	894	1,224	2,049	1,264	62	79
Scaffolds	63	7,270	50	654	4,025	2,227	787	1,362	1,611
Scaffold N50 (kb)	2,312	6	2,076	1,039	18	46	990	174	137
Contigs	63	9,506	3,717	1,548	5,249	4,276	2,047	1,424	1,687
Contig N50 (kb)	2,312	5	14	39	12	17	30	140	114
Genes	6,591	6,343	4,764	7,198	7,776	8,625	7,052	8,421	8,646
1:1 Orthologs	4291	3783	3837	4296	3956	3874	4174	3950	3958

Core**									
Short Genes^a^	102	104	109	99	69	63	88	89	85
Partial	2	551	90	18	252	201	7	4	4
Pseudo	20	0	245	10	0	0	322	0	0
Unknown function^b^	1,753	1,886	1,508	1,761	1,833	1,804	1,562	1,780	1,778
>7 exon orthologs	281	204	190	280	241	251	260	252	253
Median length (>7 exon) (aa)	477	368	340	478	500	495	462	455	443

Subtelomeres**									
Short Genes^a^	46	278	117	71	536	531	131	857	997
Partial	8	621	246	262	547	676	156	2	6
Pseudogenes	1236	3	21	978	4	6	393	11	10
Unknown function^b^	765	1328	447	437	1176	1330	734	1824	2122

a Less than 100 amino acids.

b Annotated as either ‘hypothetical protein’ or ‘conserved *Plasmodium protein*’.

** Core defined as genes that have 1–1 orthologues between *P. falciparum* 3D7 and *P. vivax* P01.

Grey columns indicate genome assemblies from ref. 7.

**Extended Data Table 2:** Samples positive for different *Plasmodium* species in the Pf3K data set

Country	Total Samples	*P. falciparum* Positive	*P. vivax* Positive	*P. malariae* Postive	*P. ovale* Positive	*P. knowlesi* Positive
The Gambia	65	65	0	0	0	0
Guinea	100	100	0	7	3	0
Thailand	148	148	11	0	0	0
Ghana	617	617	5	12	9	0
Cambodia	570	570	50	0	0	0
Mali	96	96	0	1	0	0
Bangladesh	50	50	4	0	0	0
Malawi	369	369	4	4	4	0
Vietnam	97	97	16	0	0	0
Myanmar	60	60	7	0	0	0
Laos	85	85	4	0	2	0
DR Congo	113	113	1	2	1	0
Nigeria	5	5	0	0	0	0
Senegal	137	137	0	0	1	0
GLOBAL	2512	2512	102	26	19	0

The first column shows country of origin for the different samples, with the second column showing the total number of samples collected in that country. The following five columns show the number of these samples that are positive for the different *Plasmodium* species. All samples are positive for *P. falciparum*, which is expected because all the samples were initially identified as *P. falciparum* infections. We do not see any samples positive for *P. knowlesi*, because it has a very limited geographic range and isn’t found in any of the sampled countries to our knowledge.

**Extended Data Table 3:** Genes with significant scores in same test for both *P. falciparum* and *P. reichenowi* and either *P. o. curtisi* and *P. o. wallikeri* or *P. malariae* and *P.* malariae-like

Species	Gene ID	Gene Product
*P. malariae*	PmUG01_09042600	apical membrane antigen 1
*P. malariae*	PmUG01_l1024300	conserved Plasmodium protein
*P. malariae*	PmUG01_03026800	ferrodoxin reductase-like protein
*P. malariae*	PmUG01_07042000	merozoite surface protein 1
*P. malariae*	PmUG01_08020600	ADP/ATP carrier protein
*P. malariae*	PmUG01_08045200	conserved Plasmodium protein
*P. malariae*	PmUG01_l1040300	EGF-like membrane protein
*P. o. curtisi*	PocGH01_13025000	NAD(P)H-dependent glutamate synthase

For the three population genetics measures (HKAr, K_a_/K_s_, and MK Skew), the table shows the genes that have significant values in both the *P. falciparum* and *P. reichenowi* comparison and either the *P. o. curtisi* and *P. o. wallikeri* or the *P. malariae* and *P. malariae*-like comparison.

**Extended Data Table 4:** List of potential RBP1a receptors

Human Gene ID	Annotation
HUMAN_NP_006570.1	3-beta-hydroxysteroid-Delta(8),Delta(7) isomerase
HUMAN_NP_001095937.1	Aquaporin-12b precursor
HUMAN_NP_001182010.1	Claudin-34
HUMAN_NP_000485.3	Collagen alpha-1 (XVII] chain
HUMAN_NP_001268861.1	Condensin-2 complex subunit G2 isoform a
HUMAN_NP_001073922.2	Integrator complex subunit 1
HUMAN_NP_001185744.1	Mucin-22 precursor
HUMAN_NP_001017989.2	Optic atrophy 3 protein isoform a
HUMAN_NP_072093.2	Probable G-protein coupled receptor 135
HUMAN_NP_001290402.1	Probable G-protein coupled receptor 146
HUMAN_NP_006356.1	Protein CROC-4
HUMAN_NP_001192181.1	RING finger protein 223
HUMAN_NP_612145.2	Serine palmitoyltransferase small subunit A
HUMAN_NP_001129975.1	Small integral membrane protein 24 precursor
HUMAN_NP_001277024.1	Transmembrane protein 114 isoform b
HUMAN_NP_001077059.1	Transmembrane protein 214 isoform 2
HUMAN_NP_001243758.1	Transmembrane protein 265
HUMAN_NP_003802.1	Tumor necrosis factor ligand superfamily member 9
HUMAN_NP_001258890.1	Zinc transporter ZIP1 isoform b

The first column shows the 19 transmembrane-containing human genes that are shared between humans and the common marmoset, but not with chimpanzees. As RBP1a is the RBP with the largest differences between *P. malariae* and *P. malariae*-like, these genes may represent interesting candidates for the RBP1a receptor.

**Extended Data Table 5:** Pseudogenized and deleted core genes in the two reference genomes

*P. vivax* ID	Annotation	*P. malariae*	*P. o. curtisi*
PVP01_0412100	Multidrug efflux pump	Pseudo	Pseudo
PVP01_0309300	Erythrocyte vesicle protein 1	Pseudo	
PVP01_1032500	Conserved *Plasmodium* protein, unknown function	Pseudo	
PVP01_1344900	Serine/Threonine protein phosphatase CPPED1	Pseudo	
PVP01_1407400	MORN repeat protein	Pseudo	
PVP01_1107900	6-cysteine protein (P92)	Deleted	
PVP01_1117100	Conserved *Plasmodium* protein, unknown function	Pseudo	
PVP01_0906000	WD repeat-containing protein WRAP73	Deleted	
PVP01_0929100	6-phosphofructokinase		Pseudo
PVP01_0940700	Carbonic anhydrase	Deleted	Pseudo
PVP01_1445600	Conserved *Plasmodium* protein, unknown function		Pseudo
PVP01_237400	Nucleoside Transporter 3		Pseudo
PVP01_1123700	Conserved *Plasmodium* protein, unknown function	Pseudo	Pseudo
PVP01_1246900	Biotin protein ligase		Pseudo

The first column shows the gene identifier of the *P. vivax* P01 homologue of the gene pseudogenized and/or deleted in one or more of the two reference genome assemblies. The second column is the *P. vivax* P01 annotation of that gene. The following two columns show whether the gene is functional (blank), pseudogenized (‘pseudo’) or deleted (‘deleted’).

## Supplementary Material

**Supplementary Information** is available in the online version of the paper.

Supplementary Data

## Figures and Tables

**Figure 1:**
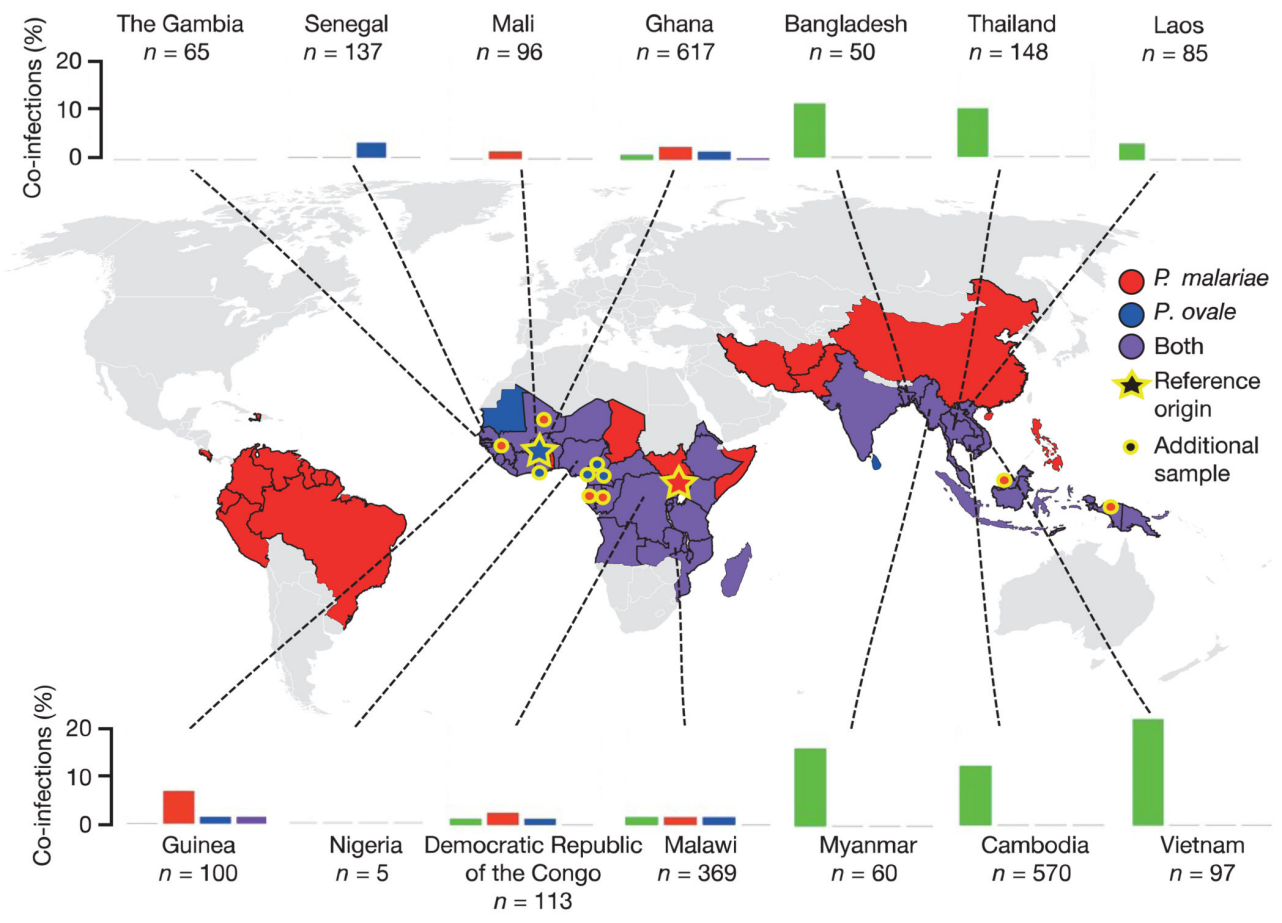
Prevalence of *P. malariae* and *P. ovale* with sample origins. Presence and absence of *P. malariae* (red), *P. ovale* (blue) or both (purple) by country on the basis of a literature review (Supplementary Information). Bar plots show proportion of *P. falciparum* infections with co-infections of *P. malariae* (red), *P. ovale* (blue), *P. vivax* (green), or two species (purple) on the basis of the Pf3K data set (Supplementary Information; Methods). Stars indicate origin of sample used for reference genome assembly and points show additional samples used. Map sourced from Wikipedia Commons (https://commons.wikimedia.org/wiki/File:BlankMap-World6.svg).

**Figure 3:**
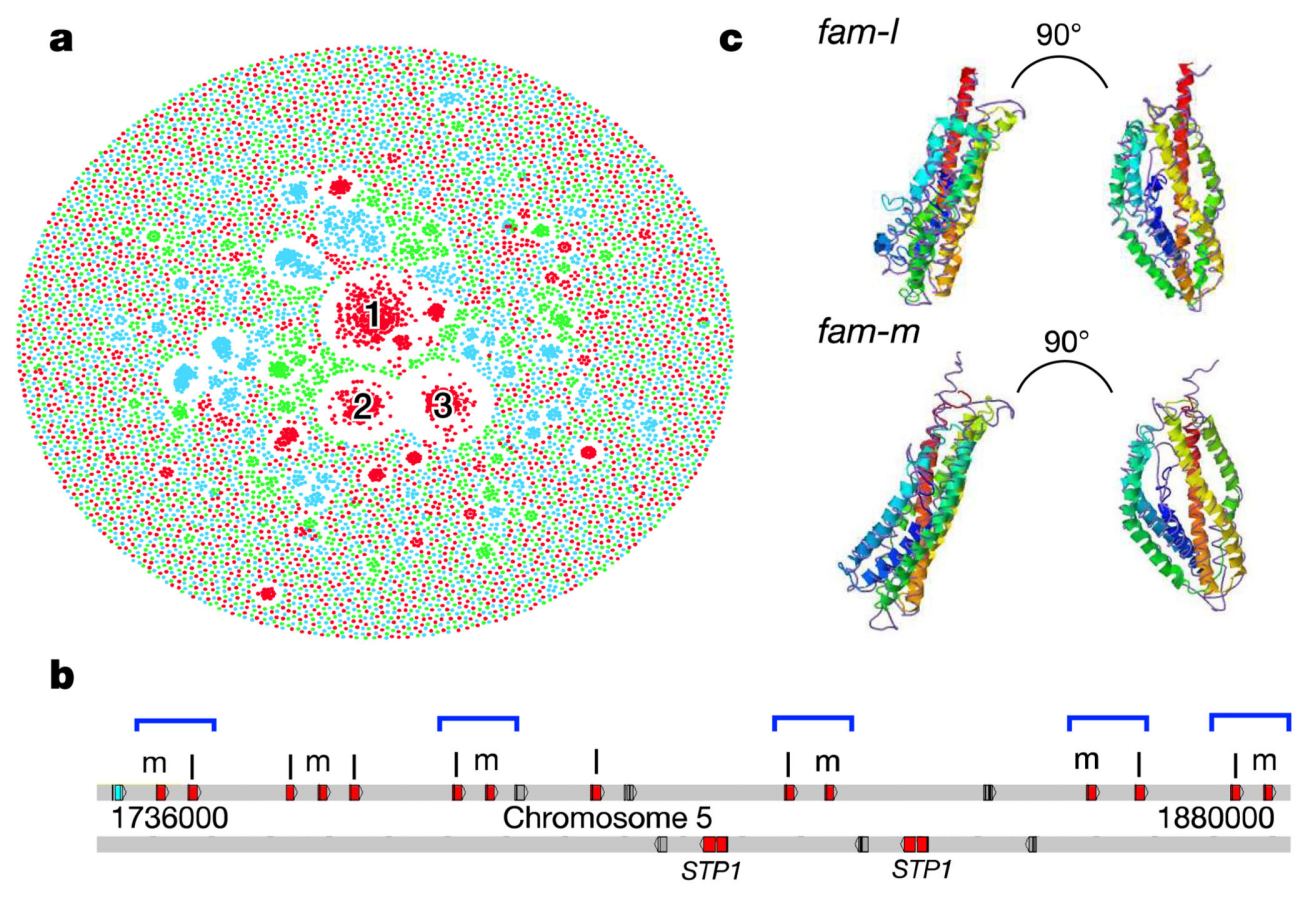
Subtelomeric gene family expansions in *P. malariae* and *P. ovale.* **a,** Gene network based on sequence similarity of all genes in *P. malariae* (red), *P. ovale* (blue), and *P. vivax* (green). Cluster 1 contains *fam-l* genes, cluster 2 contains *fam-m* genes, and cluster 3 contains *surfins* and *STP1* genes. **b,** Chromosome 5 subtelomeric localization of *fam-l* and *fam-m* genes in doublets (blue brackets) on the telomere-facing strand. Also showing pseudogenes (grey) and hypothetical gene (blue). **c,** Predicted 3D structure of *fam-l* (above) and *fam-m* (below) overlaid with the RH5 crystal structure (Purple). Right-hand images show the protein rotated to the right.

**Table 1:** Comparison of genome features of all human-infective *Plasmodium* species and *P. malariae*-like

Feature	*P. malariae*	*P. malariae-like*	*P. ovale curtisi*	*P. ovale wallikeri*	*P. falciparum*	*P. knowlesi*	*P. vivax*
Genome size (Mb)	33.6	23.7	33.5	33.5	23.3	24.4	29.1
Scaffolds^a^	14 (47)	14 (36)	14 (638)	14 (771)	14 (0)	14 (297)	14 (226)
Gaps	0	3697	894	1264	0	98	560
GC content	0.24	0.30	0.29	0.29	0.19	0.39	0.40
Gene Number*	6,540	4,764**	7,132	7,052**	5,429	5,291	6,642
Pseudogenes	623	N/A	494	N/A	153	7	154
*pir*	255	4	1,949	1,375	227	70	1,212
*var*	0	0	0	0	103	0	0
*SICAvar*	0	0	0	0	0	237	0
*STP1*	166	2	70	94	0	0	9
*tryptophan-rich antigen*	42	7	41	33	3	29	40
*ETRAMP*	8	4	7	11	15	10	10
*PHIST*	30	3	54	21	81	44	82
*fam-l*^b^	396	0	0	0	0	0	0
*fam-m*^b^	283	1	0	0	0	0	0
Nucleotide Diversity	3.2 × 10^−4^	6.5 × 10^−3^	1.9 × 10^−4^	3.7 × 10^−4^	5.7 × 10^−4^	N/A	9.9 × 10^−4^

* including pseudogenes and partial genes, excluding non-coding RNA genes.

** Non-curated gene-models.

a Unassigned contigs indicated in parentheses.

b Previously included in the *Pm-fam-a* family^7^, which consisted of all unannotated transmembrane-containing genes.
