# Supplementary material for: Plasmodium malariae and P. ovale genomes provide insights into malaria parasite evolution
Source: Nature. Author manuscript; Available in PMC 2017 Feb 25. (PMC5326575; doi:10.1038/nature21038)
Supplement: Supplementary Data [file NIHMS71595-supplement-Supplementary_Data.pdf]

doi:10.1038/nature21038

Contents

I. Co-Infections

Additional details concerning the co-infection mining, including placing the findings into context and describing the limitations of the methodology.

II. Genome Assemblies

Additional discussion on the quality of the genomes assembled for this study, as well as a comparison to available draft genomes.

Supplementary Table 1. Accession Numbers for the Assembled Genome Sequences

III. Hypnozoite Genes

Analysis and results of a systematic search for potential hypnozoite genes.

Supplementary Table 2. Putative Hypnozoite Candidate Genes

Supplementary Table 3. Conserved Genes containing Sporozoite-specific ApiAP2 motifs

IV. Phylogenetic Analyses

Discussion of sensitivity tests performed for the phylogenetic tree presented in this study.

Supplementary Table 4. Effects of Different Filtering Methods on Resulting Phylogenetic Tree

Supplementary Table 5. Correlations of molecular evolution coefficients with number of sites discarded (absolute) and proportion retained (Retained)

V. Lineage Dating

Additional details describing the assumptions made for dating the relative split of the different species.

VI. Population Genetics

SNP calling details and in-depth analysis of genes under selection in the two manually curated genomes.

Supplementary Table 6. Origin, sequencing statistics, and usage of samples in this study

Supplementary Table 7. *P. malariae* SNP calling results

Supplementary Table 8. *P. ovale* SNP calling results

Supplementary Table 9. Genes with significant scores in two or more molecular evolution coefficients

VII. RBP1a Receptor

Additional discussion about the search for a potential RBP1a receptor.

I. Co-Infections

Infections with *P. malariae* and *P. ovale* are frequently asymptomatic[1] and often have parasitaemia levels undetectable by light microscopy[2], making their study in human populations difficult and potentially thwarting efforts to eliminate them and declare any regions 'malaria free'[3]. This lack of knowledge is especially worrying because the two species are distributed widely across all malaria-endemic areas of the world[4,5] (Figure 1). *P. malariae* and *P. ovale* frequently occur as co-infections with the most common species, *P. falciparum,* and can be present in up to 5% of all clinical malaria cases[1]. In general, *P. malariae* is thought to be more common in West Africa, where reported field survey prevalences exceed 15%[6], than in other areas where it is endemic and is only thought to be present in about 1-2% of malaria cases[7]. Antagonistic interactions between *P. vivax* and *P. malariae* have been previously suggested to explain this observation[8,9]. *P. ovale* is generally thought to have low (1-2%) prevalences throughout all regions where it is endemic[9], though this may be an underestimate due to its very low parasitaemia and short duration of patent infections[9].

We conducted a literature search to document the global distribution of *P. o. curtisi, P. o. wallikeri,* and *P. malariae* (Figure 1). Due to the difficulties in distinguishing the former two, we collapsed both species into *P. ovale*. We searched pubmed (http://www.ncbi.nlm.nih.gov/pubmed) with the search terms "*Plasmodium ovale*" and "*Plasmodium malariae*" from the year 2000 onwards. We limited ourselves to

this timeframe due to the changing distribution of malaria, mostly due to ongoing control efforts. This resulted in 491 publications for *P. ovale* and 661 publications for *P. malariae,* specifying the country-specific presence or absence of either of the two species. Following this, a targeted search was conducted for countries where only one of the two species was found by searching in pubmed for the other species followed by the country name. Finally, another targeted search was performed for countries surrounded by countries where either or both of the species were found. This literature review confirms that both *P. malariae* and *P. ovale* are found throughout most malaria endemic regions, with the well documented[10] exception of *P. ovale* not being endemic in South America.

Most studies looking at *P. malariae* and *P. ovale* prevalences do so by either employing light microscopy[11], PCR probes[12], or looking at seroprevalence[2]. Differences between parasite prevalences as estimated using light microscopy and PCR methods have been documented[13], and are linked to the low parasitaemia levels of *P. malariae* and *P. ovale.* Knowing that both species are frequently observed in co-infections with *P. falciparum,* we decided to develop a new method that looks for species-specific mitochondrial SNPs in existing *P. falciparum* sequencing data (Methods). Due to the higher copy number of mitochondrial compared to nuclear DNA, we are able to identify species specific SNPs at ~10 fold lower frequencies than using nuclear markers. Using this method, we found *P. malariae* and *P. ovale* in approximately 2% of all *P. falciparum* clinical infections from the global sample collection of the Pf3K project (www.malariagen.net) (Figure 1; Extended Data Table

2). This compares to about 4% of *P. falciparum* infections being co-infections with *P. vivax* using the same method, with an average of about 10% in Southeast Asia, which is in line with known estimates[14]. We also found a number of infections containing three species. We found *P. malariae* and *P. ovale* solely in African countries, with Asian samples only containing *P. vivax* co-infections. This could in part be due to the known differences in prevalence of *P. malariae* between West Africa and other regions[9] and may be more evidence of antagonistic interactions between *P. vivax* and the other species[8]. Another explanation could be geographical differences in mitochondrial SNPs between the sampling locations in Southeast Asia and our available samples. The presence of the two species in about 2% of infections is in line with similar estimates using other detection methods[9]. However, due to the sampling bias of only looking within clinical *P. falciparum* infections and due to possible differences in mitochondrial SNPs across geographical regions, the 2% prevalence found for the two species is likely to be a significant underestimate.

II. Genome Assemblies

A 33.6 megabase (Mb) reference genome of *P. malariae* was produced from clinically isolated parasites and sequenced using Pacific BioSciences long-read sequencing technology (Methods). The assembled sequence comprised 14 super-contigs representing the 14 chromosomes, with 6 chromosome ends extending into telomeres, and a further 47 unassigned subtelomeric contigs containing an additional 11 telomeric sequences (Table 1). Using existing Illumina sequence data from two patients primarily infected with *P. falciparum*, reads were extracted

(Methods) and assembled into 33.5 Mb genomes for both *P. o. curtisi* and *P. o. wallikeri,* each assembly comprising fewer than 800 scaffolds and both being more contiguous than concurrently available alternatives[15] (Scaffold N50 *P. o. curtisi*: 1 Mb vs. 46 kb; *P. o. wallikeri*: 990 kb vs. 174 kb) (Table 1; Extended Data Table 1). The genomes are significantly larger than previously sequenced *Plasmodium* species and, like *P. vivax*, have isochore structures with a higher AT content in the subtelomeres, which comprise ~40% of the genome. In addition, a *P. malariae-like* genome was produced using Illumina sequencing from parasites isolated from a chimpanzee co-infected with *P. reichenowi*. The *P. malariae-like* genome was more fragmented than the other assemblies and its 23.7 Mb sequence misses most subtelomeric regions due to whole genome amplification bias prior to sequencing. Database accession numbers for the assembled genome sequences are reported in Supplementary Table 1.

Supplementary Table 1. Accession Numbers for the Assembled Genome Sequences

Assembly Name	Study ID	Sample ID	Contig accession	Chromosome accession
PmUG01	PRJEB2579	ERS1110315	FLRL01000001-FLRL01000047	LT594622-LT594637
PocGH01	PRJEB2579	ERS1452912	FLRI01000001-FLRI01000638	LT594582-LT594597
PowCR01	PRJEB2579	ERS1452913	FLRJ01000001-FLRJ01000771	LT594505-LT594520
PmlGA01	PRJEB2579	ERS1452911	FLRK01000001-FLRK01000035	LT594489-LT594503

In comparison to the draft genomes produced by Ansari et al[15] (Extended Data Table 1), three of the genomes assembled in the present study (PmUG01, PocGH01, PowCR01) are similar in size but significantly more contiguous. The genome of a chimpanzee-infecting species known as *P. malariae-like* (PmlGA01) is unique to the present study but lacks good coverage of the subtelomeric regions due to biased template representation introduced by the whole genome amplification process. The assembly of *P. malariae* PmUG01 is based on long reads and comprises just 63 pieces. It has no gaps and surpasses the other assemblies according to all metrics reported (Extended Data Table 1). The assemblies of the present study are more contiguous, especially in the subtelomeric regions of the genomes.

The manual curation of gene models in the present study made a clear difference to the annotation. In addition to the annotation of pseudogenes, we were able to identify approximately 10% more genes as clear 1:1 orthologues of genes in both *P. falciparum* and *P. vivax.* Indeed, in terms of this metric, other available assemblies[15] are similar to the draft assembly of *P. malariae-like.* The highly conserved genes are especially important for cross-species comparisons and analyses. Using 1:1 orthologues as indicators of genes within the conserved core regions of chromosomes, we see that our assemblies have about 20% more short genes (less than 100 codons) annotated (averages: 100 versus 82), being thereby more similar in number to those seen in *P. falciparum* (101). Multi-exon genes are notoriously difficult to annotate; looking at the number of 1:1 orthologues in the different assemblies to *P. vivax* and *P. falciparum* genes with over 7 exons (300), the genomes

of this study (excluding PmlGA01) have both 10% more 1:1 orthologues annotated and less variable median lengths between assemblies (range: 462-478 versus 368-500[15]). The large number of partial genes observed in some of the genomes assembled by Ansari et al[15] is due to the higher amount of genes truncated by contig boundaries.

The subtelomeres in *P. malariae* and *P. ovale* required significant manual curation due to the high number of pseudogenes present and the ease in which exons can mistakenly be missed during annotation. Excluding PmlGA01 that lacks most subtelomeric regions, all assemblies in the present study have significantly more genes annotated as pseudogenes than those by Ansari et al[15] (averages: 869 versus 7). The latter study also reports more short genes than our assemblies (averages: 640 versus 81), suggesting potential problems with gene models. Finally, the high gene numbers reported for the assemblies in Ansari et al[15] can largely be attributed to putative subtelomeric genes, most of which are short with no assigned function and therefore have an increased likelihood of being spurious.

III. Hypnozoite Genes

Both *P. ovale* species are able to form hypnozoites, similar to *P. vivax*[16] and the simian-infective *P. cynomolgi*[17]. Of 64 genes exclusive to these hypnozoite-forming species (Methods), two genes do not belong to subtelomeric gene families, encode proteins with transmembrane domains and have orthologues expressed in *P. vivax*

sporozoites[18] (Supplementary Table 2). One of the two genes has weak similarity to the *P. falciparum* ring-exported protein 4 gene.

Supplementary Table 2. Putative Hypnozoite Candidate Genes

PVP01 Annotation	*P. vivax*	*P. cynomolgi*	*P. o. curtisi*
Ring-exported protein 4*	PVP01_0623900	Pcyb_063280	PocGH01_00129400
Conserved Plasmodium protein	PVP01_1402600	Pcyb_141110	PocGH01_00080600

*not in the same orthologous group as *P. falciparum* REX4

Looking at genes previously suggested to be involved in hypnozoite formation[17], we did not find *P. o. curtisi* orthologues of the three genes shared exclusively by *P. vivax* and *P. cynomolgi* that contain sporozoite-specific ApiAP2 motifs. However, we do find such motifs in six out of nine dormancy related genes[17], including Ran (PocGH01_09023900) previously identified in a *P. vivax* screen for potential hypnozoite genes[16] (Supplementary Table 3).

Supplementary Table 3. Conserved Genes containing Sporozoite-specific ApiAP2 motifs

P. vivax PVP01	*P. o. curtisi*	Annotation
PVP01_0726200	PocGH01_07035100	Serine/threonine protein phosphatase 4
PVP01_0825700	PocGH01_08034100	Serine/threonine protein phosphatase 6
PVP01_0918300	PocGH01_09023900	GTP-binding nuclear protein Ran/TC4
PVP01_1115000	PocGH01_00015700	Protein kinase 5
PVP01_1205500	PocGH01_12013900	Tyrosine kinase-like protein
PVP01_1257700	PocGH01_12063800	Transcription factor IIIb subunit

Dormancy-related genes identified as containing a sporozoite-specific ApiAP2 motif in their 1kb 5' upstream region in *P. vivax, P. cynomolgy*[17]*,* and *P. o. curtisi.*

IV. Phylogenetic Analyses

A number of conflicting phylogenetic trees of the *Plasmodium* genus have been published that differ in their placement of *P. ovale* and *P. malariae.* The two most commonly reported topologies either place *P. ovale* as a sister taxon to the rodent malaria parasites[19] (Tree A) or as an outgroup to *P. malariae* and *P. vivax*[15,20,21] (Tree B). The same studies also place *P. malariae* as either a distant outgroup to both the rodent malaria parasites and the *P. vivax* clade[19], or as being more closely related to *P. vivax* than *P. ovale,* with *P. malariae* thereby being a close outgroup to the primate infective clade[15,21]. A recent study using draft genome sequences supported Tree B[15], while the phylogenetic tree presented in this study supports the Tree A topology (Figure 2).

Following orthologue assignment using BLASTP[22] and OrthoMCL[23], amino acid sequences of 1000 core genes from 12 *Plasmodium* species (*P. gallinaceum*[24], *P. falciparum*[25], *P. reichenowi*[26], *P. knowlesi*[27], *P. vivax*[28], *P. cynomolgi*[17], *P. chabaudi*[29], *P. berghei*[30], and the four assemblies produced in this study) were aligned using MUSCLE[31]. The alignments were cleaned using GBlocks[32] with default parameters to remove non-informative and gapped alignment columns. The cleaned non-zero length alignments were then concatenated. This resulted in an alignment of 421,988 amino acid sites per species, the largest alignment including *P. malariae* and *P. ovale* used to date[15].

We determined the sensitivity of the tree topology to different parameters and tree-building algorithms, we analysed the 1000 core gene alignment used for the tree in Figure 2 using a number of different algorithms for phylogenetic inference. We utilized different tree-building softwares, including RAxML[33] (see below), PhyloBayes[34] (using ratecat, cat, and uni models), and PhyML[35] (LG model with optimized site rates), all resulting in Tree A. We also used a number of different amino acid substitution models implemented within RAxML[33] version 8.2.4., including JTT, LG, LG4M, LG4X, GTR_unlinked, GTR, and DAYHOFF. All these models were tested with both CAT and GAMMA site distribution rates. All substitution models resulted in Tree A. In addition to this, we calculated the optimal substitution model for each gene partition by running RAxML[33] for each gene separately using all implemented substitution models (minimum AIC). Using this substitution model optimized partitioned alignment we still generated Tree A. In order to determine whether the conflicting topology would be a local optimum and was therefore not found using our other approaches, we used Tree B as the starting tree using RAxML[33] with a PROTGAMMAJTT substitution model. This approach still converged on Tree A, indicating that our alignment does not support the Tree B topology.

We performed bootstrapping as implemented within RAxML[36], obtaining very good bootstrap support for all nodes (Figure 2). We also tested the RAxML '-f S' parameter with a window size of 10 and Tree A as the reference tree. This computes phylogenetic signal strengths for each site in the alignment using a leave-one-out approach[37]. We used this output to filter away the top 5% of sites that either

strongly supported or strongly did not support Tree A. Using this trimmed alignment, RAxML still produced Tree A, indicating that the phylogenetic signal is not driven by a small subset of sites. Finally, we generated maximum likelihood trees using RAxML[33] with an LG4X model for additional GBlocks[32] trimmed alignments consisting of 200, 500, and 3298 orthologous genes (the latter being all genes with 1-1 orthologs across all 12 species). All alignments resulted in Tree A with good bootstrap support[36].

We generated separate phylogenetic trees using RAxML for each gene in the 1000 orthologous gene alignment using their optimal substitution models (see above). The consensus tree, as calculated using RAxML[33], of these gene-specific trees was Tree A. Most nodes were supported by the majority of gene trees (>50%). The two nodes that differ between Tree A and Tree B are less well supported. Only 25% of genes support the placement of *P. malariae* as the distant outgroup, while 38% support *P. ovale* branching off with the rodent malaria parasites. While these percentages may seem low, a lower proportion of genes support Tree B, namely only 19% of genes support the placement of *P. ovale* as an outgroup to *P. malariae* and *P. vivax,* while 24% place *P. malariae* between *P. ovale* and *P. vivax.* This shows that there is significant heterogeneity in the phylogenetic signal present and that these particular nodes are difficult to resolve. We however show that a larger proportion of genes- and the strongest signals when alignments of all genes are concatenated- support Tree A than support Tree B.

In order to determine the effect of alignment filtering on the resulting tree, we constructed phylogenetic trees using RAxML, with the JTT amino acid substitution model as in Ansari, et al. [15], using a number of different alignment trimming strategies. This included no trimming, trimming using Gblocks[32] default parameters (as above), loosening the Gblocks[32] parameters by allowing some gapped sites (if in less than 50% of sequences) and allowing smaller synteny blocks (down to 2 sites), as well as all three TrimAl[38] preset options (nogap, strict, strictplus), as used in Ansari, et al. [15] (Supplementary Table 4).

Supplementary Table 4. Effects of Different Filtering Methods on Resulting Phylogenetic Tree

Trimming Method	Retained Amino Acids	Phylogenetic Tree
Untrimmed	1,012,857	Tree C
GBlocks (default param.)	421,988	Tree A
GBlocks (liberal param.)	480,453	Tree C
TrimAl (nogap)	569,568	Tree C
TrimAl (strict)	450,571	Tree A
TrimAl (strictplus)	418,240	Tree A

Of the six trimming methods, the three most stringent filtering methods all resulted in Tree A. The other three trees labeled as 'Tree C' were identical, not placing *P. ovale* as a sister taxon with the rodent malaria parasites but still differing from Tree B by placing *P. malariae* in the same position as Tree A. We were therefore unable to generate Tree B using our alignment. We show however that the stringency of the alignment filtering can severely impact the placement of *P. ovale.* The more stringent the filtering, the more likely *P. ovale* is placed as a sister taxon to the rodent malaria species.

Due to the impact that filtering has on determining the topology, we investigated whether the filtering performed using GBlocks[32] with default parameters is appropriate. We correlated (Pearson's correlation coefficient, r) the absolute number of sites removed (Absolute) and the proportion of the gene sequence retained (Retained) with a number of molecular evolution selection coefficients (HKAr, Ka/Ks, MK p-value) calculated for three species-species comparisons (see below) for each gene. Note that these statistics were calculated using variant calls directly from mapped reads, and so should be relatively robust to alignment quality itself. Supplementary Table 5 shows the Pearson's correlation coefficient (r) and Bonferroni adjusted p-values for those correlations:

Supplementary Table 5. Correlations of molecular evolution coefficients with number of sites discarded (absolute) and proportion retained (Retained)

	HKAr		Ka/Ks		MK (p-value)	
	Absolute	Retained	Absolute	Retained	Absolute	Retained
P. o. curtisi/ P. o. wallikeri	0.099 *	-0.066	0.324 ***	-0.478 ***	-0.179 ***	0.025
P. malariae/ P. malariae-like	0.139 **	-0.088	0.312 ***	-0.365 ***	-0.178 ***	0.090
P. falciparum/ P. reichenowi[26]	0.094	-0.115 *	0.454 ***	-0.554 ***	-0.194 ***	0.107 *

* $p < 0.1$, ** $p < 0.01$, *** $p < 0.001$

The table shows that there is a strong correlation between Ka/Ks and MK for all three comparisons with the number of sites removed from each gene. The strong positive correlation for Ka/Ks indicates that Ka/Ks is higher in genes where more of

the gene sequence is removed. The strong negative correlation for MK (p-value) indicates that the p-values tend to be lower (i.e. more likely to be significant) in genes where more of the gene sequence is removed. Hence, genes that seem to be under significant selective pressures tend to be filtered more heavily using GBlocks[32]. This is as expected, as Ka/Ks measures are increased by bad alignments. This means that the filtered alignment consists of a larger proportion of neutrally evolving sites, which are more informative for phylogenetic inference.

We performed a GO term enrichment analysis[39] by looking at the top 10% of genes that were filtered either the most or the least by GBlocks[32]. We find a very strong enrichment for 'GO:0006412: translation' ($p < 6e\text{-}07$) in the highly filtered genes, in addition to a number of ribosomal GO terms: 'GO:0022625: cytosolic large ribosomal subunit' ($p < 0.001$) and 'GO:0022627: cytosolic small ribosomal subunit' ($p < 0.001$). We do not see enriched GO terms in the genes that were not filtered much. Ribosomal genes are often either extremely conserved or highly variable, making them difficult to align and they were therefore filtered away by GBlocks[32]. Many of the genes that we filtered away using GBlocks[32], including ribosomal genes and surface antigens such as *ama1*, were previously included in a manually selected genelist that generated Tree B[15].

The genes chosen by Ansari, et al.[15] are enriched for those with poorer-quality sequence alignments and showing signals of selection. Our alignment filtering approach reveals a more robust signal for an alternative topology (Tree A). In any

case, we note that even without filtering, a comprehensive analysis of one-to-one orthologs between *Plasmodium* species does not support Tree B, but agrees with our preferred tree except in the placement of *P. ovale*.

V. Lineage Dating

We estimated the time of divergence for the four species using a Bayesian inference tool, G-PhoCS[40]. Absolute divergence time estimates are inherently uncertain due to mutation rate and generation time assumptions, and we therefore scaled these parameters to date the *P. falciparum* and *P. reichenowi* split using G-PhoCS to 4 million years ago (MYA), as previously published (3.0 - 5.5MYA)[41]. Testing a number of different generation time and mutation rate estimates in order to optimize the *P. falciparum*/*P. reichenowi* split to 4 million years ago as estimated previously[41], we found a mutation rate of 3.8×10^{-10} SNPs/site/lifecycle[42] and a generation time of 65 days[43] to generate this previously published date[41]. Assuming that the mutation rates and generation times are similar for *P. ovale* and *P. falciparum,* we find that the relative split of the two *P. ovale* species is about 5 times earlier than the split of *P. falciparum* and *P. reichenowi.* Using the same parameters as for the *Laverania* split, we thereby date the divergence of the two *P. ovale* subspecies to approximately 20.3MYA. Using the same mutation rate and a 50% longer generation time to account for the longer intra-erythrocytic cycle (100 days), we date the split of *P. malariae* from *P. malariae-like* to ~3.5MYA. Due to being based on non-coding elements, this divergence time is not proportional to the branch lengths of the phylogenomic tree (Figure 2) where coding regions were used.

VI. Population Genetics

Using four additional *P. malariae* samples, two additional *P. o. curtisi* samples and two *P. malariae-like* and *P. o. wallikeri* samples each, we investigated differences in selection pressures between two species that diverged based on host differences (*P. malariae* and *P. malariae-like*), and two species that supposedly diverged within the same host (*P. o. curtisi* and *P. o. wallikeri*). Supplementary Table 6 shows all samples sequenced for this study and their uses throughout.

Supplementary Table 6. Origin, sequencing statistics, and usage of samples in this study

Species	Sample ID	Accession Number	Origin	Sequencing Platform	Read Length	Usage	Library type
P. malariae	PmUG01	ERS1110315	Uganda	PacBio RS II P6/C4	N/A	Reference Genome	Size-selected
	PmMY01	ERS1110317	Malaysia	Illumina MiSeq v2<br>Illumina HiSeq 2000 v4	150bp PE<br>125bp PE	SNP Calling	PCR Amplification free
	PmID01	ERS1110321	Papua Indonesia	Illumina MiSeq v2<br>Illumina HiSeq 2000 v4	150bp PE<br>125bp PE	SNP Calling	PCR Amplification free
	PmMA01	ERS1110325	Mali	Illumina MiSeq v2	150bp PE	SNP Calling	Standard
	PmGN01	ERS567899	Guinea	Illumina MiSeq v2	150bp PE<br>250bp PE	SNP Calling	Standard
P. malariae -like	PmlGA01	ERS1452911	Gabon	Illumina MiSeq v2	150bp PE	Draft Genome	WGA* - PCR Amplification free
	PmlGA02	ERS434565	Gabon	Illumina MiSeq v2	150bp PE	SNP Calling	WGA* - PCR Amplification free
P. ovale curtisi	PocGH01	ERS1452912	Ghana	Illumina HiSeq 2000 v3	100bp PE	Reference Genome	Standard
	PocGH02	ERS360497	Ghana	Illumina HiSeq 2000 v3	100bp PE	SNP Calling	Standard
	PocCR01	ERS418861	Cameroon	Illumina HiSeq 2000 v3	100bp PE	SNP Calling	Standard
P. ovale wallikeri	PowCR01	ERS1452913	Cameroon	Illumina HiSeq 2000 v3	100bp PE	Draft Genome	Standard
	PowCR02	ERS418932	Cameroon	Illumina HiSeq 2000 v3	100bp PE	SNP Calling	Standard

* WGA: Whole Genome Amplification

GATK's UnifiedGenotyper[44] was used to call single nucleotide polymorphisms (SNPs) and, following filtering (Methods), we retained 230,881 SNPs in *P. malariae* and 1,462,486 SNPs in *P. ovale* (Supplementary Table 7 and 8). Excluding subtelomeric regions, the pairwise nucleotide diversity between the different *P. malariae* samples is 3.2 x 10^{-4} and for the *P. o. curtisi* samples it is 1.9 x 10^{-4}, which are lower than the estimates we obtained for *P. vivax* (9.9 x 10^{-4}) and *P. falciparum* (5.7 x 10^{-4}) using the same methodology (Table 1). The nucleotide diversity for *P. malariae-like* is 6.5 x 10^{-3}. Interestingly, the nucleotide diversity of *P. o. wallikeri* (3.7 x 10^{-4}) appears to be much higher than that of *P. o. curtisi,* though this is difficult to confirm due to low sample numbers.

Supplementary Table 7. *P. malariae* SNP calling results

	P. malariae				*P. malariae-like*	
	PmMY01	PmID01	PmMA01	PmGN01	PmlGA01	PmlGA02
Raw SNPs	218334	164541	173028	239655	458790	211686
- Private	48094	19475	25901	66377	260540	68793
- Ref	712758	696634	706817	737236	386915	261042
- Missing*	50394	86900	74936	28776	165813	415250
Filtered SNPs	8970	8589	7742	7878	161551	140113
- Private	2149	2066	1908	2066	77781	56571
- Ref	221923	222247	223058	223003	69466	90571
- Missing*	0	0	0	0	0	0

*sites at which the sample has no coverage

SNP calling results based on mapping all *P. malariae* and *P. malariae-like* samples against the PmUG01 PacBio reference genome assembly. The raw SNPs are the total number of SNPs that we call using GATK default parameters in the different samples. Of these raw SNPs, some are exclusive to a certain sample (Private), are identical to the reference genome (Ref), or there is no coverage and therefore no SNP call could be made (Missing). The same information is also shown for the filtered SNPs, which were filtered according to a number of different parameters (Methods).

doi:10.1038/nature21038

Supplementary Table 8. *P. ovale* SNP calling results

	P. o. curtisi		*P. o. wallikeri*	
	PocGH02	PocCR01	PowCR01	PowCR02
Raw SNPs	171465	277978	2139946	1881088
- Private	36487	99083	333727	83609
- Ref	2287008	2249682	693405	674071
- Missing*	84743	72495	104013	149166
Filtered SNPs	29099	46695	1415164	1410434
- Private	6162	16026	21081	16699
- Ref	1433387	1416042	45978	50845
- Missing*	0	0	0	0

*sites at which the sample has no coverage

SNP calling results based on mapping all *P. o. curtisi* and *P. o. wallikeri* samples against the PocGH01 Illumina reference genome assembly. The raw SNPs are the total number of SNPs that we call using GATK default parameters in the different samples. Of these raw SNPs, some are exclusive to a certain sample (Private), are identical to the reference genome (Ref), or there is no coverage and therefore no SNP call could be made (Missing). The same information is also shown for the filtered SNPs, which were filtered according to a number of different parameters (Methods).

We calculated a number of selection measures for every core gene with 5 or more nucleotide substitutions and which had identifiable orthologs in *P. falciparum* and *P. vivax* (2,343 genes in *P. malariae,* 4,070 genes in *P. o. curtisi*), including the Hudson-Kreitman-Aguade ratio (HKAr)[45], which is the ratio of interspecific nucleotide divergence to intraspecific polymorphisms (*ie.* diversifying selection), Ka/Ks[46], to look for an enriched number of nonsynonymous differences compared to synonymous differences (*ie.* positive selection), and the McDonald Kreitman (MK) Skew[47], a measure of an excess of maintained nonsynonymous polymorphisms (*ie.* balancing selection). As expected by the different divergence times and therefore different interspecific nucleotide divergences, we find a high HKAr (> 0.15) in 6.1% of genes (142/2,343) in *P. malariae* but significantly fewer (0.6%; 26/4,070) in *P. o. curtisi* (2-sample test for equality of proportions, $p < 0.001$). We see more genes

under significant balancing selection in *P. malariae* (17/2,343, 0.7%) than in *P. o. curtisi* (4/4,070, 0.1%) ($p < 0.001$). Additionally, more genes are under strong positive selection (Ka/Ks > 2) in *P. malariae* (131/2,343, 5.6%) than in *P. ovale* (63/4,070, 1.4%) ($p < 0.001$).

Looking at specific genes under selection, we identify similar genes in the *P. malariae*/*P. malariae-like* test as in an earlier *P. falciparum*/*P. reichenowi* study[26], hinting at conserved selection pressures in speciation between human and chimpanzee hosts. A number of genes have high HKAr values in both comparisons, including *msp1* and genes such as *EGF-like membrane protein*, f*errodoxin reductase-like protein* and an *ADP/ATP carrier protein* gene. Two blood stage genes have significant MK skews for both comparisons, including a conserved protein of unknown function and *ama1*. Amongst genes with significant selection coefficients in both comparisons Gene Ontology (GO) annotations of 'pathogenesis' and 'entry into/exit from host cell' are significantly enriched. Similarly, a number of blood stage genes are found to have both high HKAr and significant MK skews, including *msp1*, *msp9*, and *formin-1*, all of which are known to be important in invasion and also have the same GO terms enriched.

Supplementary Table 9. Genes with significant scores in two or more molecular evolution coefficients

Species	Gene ID	Gene Product
P. malariae	PmUG01_05040800	CyRPA
P. malariae	PmUG01_07023900	alkaline phosphatase
P. malariae	PmUG01_13030900	transcription factor with AP2 domain
P. malariae	PmUG01_14040200	conserved Plasmodium protein
P. malariae	PmUG01_14019500	conserved Plasmodium protein
P. malariae	PmUG01_12012900	conserved Plasmodium protein
P. malariae	PmUG01_07042000	merozoite surface protein 1
P. malariae	PmUG01_10013600	formin 1
P. malariae	PmUG01_13030700	rRNA (adenosine-2'-O-)-methyltransferase
P. malariae	PmUG01_14062900	merozoite surface protein 9

For the three population genetics measures (HKAr, Ka/Ks, and MK Skew), the table shows the genes that have significant values in two or more of these measures. These genes therefore represent genes under significant selection pressures.

We do not see any significant sharing of selection pressures for the two *P. ovale* species with *P. falciparum/P. reichenowi,* besides an NAD(P)H-dependent glutamate synthase which has a significant MK skew in both comparisons. *P. ovale* genes with significant HKAr values include a number of transporters, including a homolog of an ABC transporter (*mrp2*), with GO terms enriched for 'drug transmembrane transport' and 'intracellular transport'. However, the five genes with the highest HKAr are all gametocyte and ookinete genes, including among others a transcription initiation factor TFIID and a mago nashi homolog protein, the latter potentially being involved in sex determination[48]. We also find that genes with low Ka/Ks (<0.5) and very high Ks/Ks (>2.5) are enriched for gametocyte genes (hypergeometric test, $p < 0.0001$ and $p < 0.001$ respectively) (Extended Data Fig 3e). Genes with high Ka/Ks values that are gametocyte-associated are enriched for genes

of unknown function (hypergeometric test, $p < 0.001$), suggesting important novel *Plasmodium* biology. Of the four genes with significant MK skews in *P. o. curtisi*, one is a kelch protein while the others are involved in 'DNA replication' and 'Telomere maintenance'. These results hint at a number of possible divergences between the two *P. ovale* species, including possible differences in drug susceptibility, changes in gametocyte genes that may have enabled speciation, while differences in DNA replication may possibly be linked to the different relapse times.

VII. RBP1a Receptor

One of the genes with the highest Ka/Ks in the *P. malariae/P. malariae-like* comparison is *RBP1a*, which has 37 nonsynonymous fixed differences between the two species and only 6 synonymous fixed differences. The other two intact RBPs are much more conserved. Knowing that *P. malariae* also infects new world monkeys (where it is known as *P. brasilianum*)[49], we might suppose that the receptor for *RBP1a* may be conserved between humans and New World monkeys, but not with chimpanzees. We identified 19 human genes encoding transmembrane proteins that form 1-1 orthologous OrthoMCL[23] clusters between humans and the common marmoset but not with chimpanzees (Extended Data Table 4; Methods). These may present a useful list of potential *RBP1a* receptors and include a mucin-22 precursor and an aquaporin 12b precursor.

Supplementary References

1 Roucher, C., Rogier, C., Sokhna, C., Tall, A. & Trape, J. F. A 20-year longitudinal study of Plasmodium ovale and Plasmodium malariae prevalence and morbidity in a West African population. *PLoS One* **9**, e87169, doi:10.1371/journal.pone.0087169 (2014).

2 Doderer-Lang, C. *et al.* The ears of the African elephant: unexpected high seroprevalence of Plasmodium ovale and Plasmodium malariae in healthy populations in Western Africa. *Malar J* **13**, 240, doi:10.1186/1475-2875-13-240 (2014).

3 Bousema, T., Okell, L., Felger, I. & Drakeley, C. Asymptomatic malaria infections: detectability, transmissibility and public health relevance. *Nat Rev Microbiol* **12**, 833-840, doi:10.1038/nrmicro3364 (2014).

4 Collins, W. E. & Jeffery, G. M. Plasmodium ovale: parasite and disease. *Clin Microbiol Rev* **18**, 570-581, doi:10.1128/CMR.18.3.570-581.2005 (2005).

5 Collins, W. E. & Jeffery, G. M. Plasmodium malariae: parasite and disease. *Clin Microbiol Rev* **20**, 579-592, doi:10.1128/CMR.00027-07 (2007).

6 Trape, J. F. *et al.* The Dielmo project: a longitudinal study of natural malaria infection and the mechanisms of protective immunity in a community living in a holoendemic area of Senegal. *Am J Trop Med Hyg* **51**, 123-137 (1994).

7 Kawamoto, F., Liu, Q., Ferreira, M. U. & Tantular, I. S. How prevalent are Plasmodium ovale and P. malariae in East Asia? *Parasitol Today* **15**, 422-426 (1999).

8 Smith, T. *et al.* Prospective risk of morbidity in relation to malaria infection in an area of high endemicity of multiple species of Plasmodium. *Am J Trop Med Hyg* **64**, 262-267 (2001).

9 Mueller, I., Zimmerman, P. A. & Reeder, J. C. Plasmodium malariae and Plasmodium ovale--the "bashful" malaria parasites. *Trends Parasitol* **23**, 278-283, doi:10.1016/j.pt.2007.04.009 (2007).

10 Castellanos, M. E. *et al.* First imported Plasmodium ovale malaria in Central America: case report of a Guatemalan soldier and a call to improve its accurate diagnosis. *Mil Med Res* **2**, 3, doi:10.1186/s40779-015-0030-9 (2015).

11 Ghosh, S. K. & Yadav, R. S. Naturally acquired concomitant infections of bancroftian filariasis and human plasmodia in Orissa. *Indian J Malariol* **32**, 32-36 (1995).

12 Zhou, M. *et al.* High prevalence of Plasmodium malariae and Plasmodium ovale in malaria patients along the Thai-Myanmar border, as revealed by acridine orange staining and PCR-based diagnoses. *Trop Med Int Health* **3**, 304-312 (1998).

13 Lek, D. *et al.* National Malaria Prevalence in Cambodia: Microscopy Versus Polymerase Chain Reaction Estimates. *Am J Trop Med Hyg* **95**, 588-594, doi:10.4269/ajtmh.15-0908 (2016).

14 Tripura, R. *et al.* Persistent Plasmodium falciparum and Plasmodium vivax infections in a western Cambodian population: implications for prevention, treatment and elimination strategies. *Malar J* **15**, 181, doi:10.1186/s12936-016-1224-7 (2016).

15 Ansari, H. R. *et al.* Genome-scale comparison of expanded gene families in Plasmodium ovale wallikeri and Plasmodium ovale curtisi with Plasmodium malariae and with other Plasmodium species. *Int J Parasitol*, doi:10.1016/j.ijpara.2016.05.009 (2016).

16 Carlton, J. M. *et al.* Comparative genomics of the neglected human malaria parasite Plasmodium vivax. *Nature* **455**, 757-763, doi:10.1038/nature07327 (2008).

17 Tachibana, S. *et al.* Plasmodium cynomolgi genome sequences provide insight into Plasmodium vivax and the monkey malaria clade. *Nat Genet* **44**, 1051-1055, doi:10.1038/ng.2375 (2012).

18 Westenberger, S. J. *et al.* A systems-based analysis of Plasmodium vivax lifecycle transcription from human to mosquito. *PLoS Negl Trop Dis* **4**, e653, doi:10.1371/journal.pntd.0000653 (2010).

19 Arisue, N. *et al.* The Plasmodium apicoplast genome: conserved structure and close relationship of P. ovale to rodent malaria parasites. *Mol Biol Evol* **29**, 2095-2099, doi:10.1093/molbev/mss082 (2012).

20 Sutherland, C. J. *et al.* Two nonrecombining sympatric forms of the human malaria parasite Plasmodium ovale occur globally. *J Infect Dis* **201**, 1544-1550, doi:10.1086/652240 (2010).

21 Schaer, J. *et al.* High diversity of West African bat malaria parasites and a tight link with rodent Plasmodium taxa. *P Natl Acad Sci USA* **110**, 17415-17419, doi:10.1073/pnas.1311016110 (2013).

22 Altschul, S. F. *et al.* Gapped BLAST and PSI-BLAST: a new generation of protein database search programs. *Nucleic Acids Res* **25**, 3389-3402 (1997).

23 Li, L., Stoeckert, C. J., Jr. & Roos, D. S. OrthoMCL: identification of ortholog groups for eukaryotic genomes. *Genome Res* **13**, 2178-2189, doi:10.1101/gr.1224503 (2003).

24 Boehme, U. *et al.* Complete avian malaria parasite genomes reveal host-specific parasite evolution in birds and mammals. *bioRxiv*, doi:10.1101/086504 (2016).

25 Gardner, M. J. *et al.* Genome sequence of the human malaria parasite Plasmodium falciparum. *Nature* **419**, 498-511, doi:10.1038/nature01097 (2002).

26 Otto, T. D. *et al.* Genome sequencing of chimpanzee malaria parasites reveals possible pathways of adaptation to human hosts. *Nat Commun* **5**, 4754, doi:10.1038/ncomms5754 (2014).

27 Pain, A. *et al.* The genome of the simian and human malaria parasite Plasmodium knowlesi. *Nature* **455**, 799-803, doi:10.1038/nature07306 (2008).

28 Auburn, S. *et al.* A new Plasmodium vivax reference sequence with improved assembly of the subtelomeres reveals an abundance of pir genes. *Wellcome Open Res* **1**, 4, doi:10.12688/wellcomeopenres.9876.1 (2016).

29 Otto, T. D. *et al.* A comprehensive evaluation of rodent malaria parasite genomes and gene expression. *BMC Biol* **12**, 86, doi:10.1186/s12915-014-0086-0 (2014).
30 Fougere, A. *et al.* Variant Exported Blood-Stage Proteins Encoded by Plasmodium Multigene Families Are Expressed in Liver Stages Where They Are Exported into the Parasitophorous Vacuole. *PLoS Pathog* **12**, e1005917, doi:10.1371/journal.ppat.1005917 (2016).
31 Edgar, R. C. MUSCLE: multiple sequence alignment with high accuracy and high throughput. *Nucleic Acids Res* **32**, 1792-1797, doi:10.1093/nar/gkh340 (2004).
32 Talavera, G. & Castresana, J. Improvement of phylogenies after removing divergent and ambiguously aligned blocks from protein sequence alignments. *Syst Biol* **56**, 564-577, doi:10.1080/10635150701472164 (2007).
33 Stamatakis, A., Ludwig, T. & Meier, H. RAxML-III: a fast program for maximum likelihood-based inference of large phylogenetic trees. *Bioinformatics* **21**, 456-463, doi:10.1093/bioinformatics/bti191 (2005).
34 Lartillot, N., Lepage, T. & Blanquart, S. PhyloBayes 3: a Bayesian software package for phylogenetic reconstruction and molecular dating. *Bioinformatics* **25**, 2286-2288, doi:10.1093/bioinformatics/btp368 (2009).
35 Guindon, S. *et al.* New algorithms and methods to estimate maximum-likelihood phylogenies: assessing the performance of PhyML 3.0. *Syst Biol* **59**, 307-321, doi:10.1093/sysbio/syq010 (2010).
36 Stamatakis, A., Hoover, P. & Rougemont, J. A rapid bootstrap algorithm for the RAxML Web servers. *Syst Biol* **57**, 758-771, doi:10.1080/10635150802429642 (2008).
37 Berger, S. A., Krompass, D. & Stamatakis, A. Performance, accuracy, and Web server for evolutionary placement of short sequence reads under maximum likelihood. *Syst Biol* **60**, 291-302, doi:10.1093/sysbio/syr010 (2011).
38 Capella-Gutierrez, S., Silla-Martinez, J. M. & Gabaldon, T. trimAl: a tool for automated alignment trimming in large-scale phylogenetic analyses. *Bioinformatics* **25**, 1972-1973, doi:10.1093/bioinformatics/btp348 (2009).
39 Alexa, A. R., J. topGO: Enrichment Analysis for Gene Ontology. *R package version 2.24.0* (2016).
40 Gronau, I., Hubisz, M. J., Gulko, B., Danko, C. G. & Siepel, A. Bayesian inference of ancient human demography from individual genome sequences. *Nat Genet* **43**, 1031-1034, doi:10.1038/ng.937 (2011).
41 Silva, J. C., Egan, A., Arze, C., Spouge, J. L. & Harris, D. G. A new method for estimating species age supports the coexistence of malaria parasites and their Mammalian hosts. *Mol Biol Evol* **32**, 1354-1364, doi:10.1093/molbev/msv005 (2015).
42 Claessens, A. *et al.* Generation of antigenic diversity in Plasmodium falciparum by structured rearrangement of Var genes during mitosis. *PLoS Genet* **10**, e1004812, doi:10.1371/journal.pgen.1004812 (2014).

43 Daniels, R. F. *et al.* Modeling malaria genomics reveals transmission decline and rebound in Senegal. *Proc Natl Acad Sci U S A* **112**, 7067-7072, doi:10.1073/pnas.1505691112 (2015).

44 McKenna, A. *et al.* The Genome Analysis Toolkit: a MapReduce framework for analyzing next-generation DNA sequencing data. *Genome Res* **20**, 1297-1303, doi:10.1101/gr.107524.110 (2010).

45 Innan, H. Modified Hudson-Kreitman-Aguade test and two-dimensional evaluation of neutrality tests. *Genetics* **173**, 1725-1733, doi:10.1534/genetics.106.056242 (2006).

46 Nekrutenko, A., Makova, K. D. & Li, W. H. The K(A)/K(S) ratio test for assessing the protein-coding potential of genomic regions: an empirical and simulation study. *Genome Res* **12**, 198-202, doi:10.1101/gr.200901 (2002).

47 Kreitman, M. & Hudson, R. R. Inferring the evolutionary histories of the Adh and Adh-dup loci in Drosophila melanogaster from patterns of polymorphism and divergence. *Genetics* **127**, 565-582 (1991).

48 Li, W., Boswell, R. & Wood, W. B. mag-1, a homolog of Drosophila mago nashi, regulates hermaphrodite germ-line sex determination in Caenorhabditis elegans. *Dev Biol* **218**, 172-182, doi:10.1006/dbio.1999.9593 (2000).

49 Lalremruata, A. *et al.* Natural infection of Plasmodium brasilianum in humans: Man and monkey share quartan malaria parasites in the Venezuelan Amazon. *EBioMedicine* **2**, 1186-1192, doi:10.1016/j.ebiom.2015.07.033 (2015).
